# Digital Health Technology Adoption Among Chinese Physicians: Latent Profile Analysis and Cross-Sectional Study

**DOI:** 10.2196/77840

**Published:** 2025-11-26

**Authors:** Zhichao Wang, Jiao Lu, Zhongliang Zhou, Guanping Liu, Xiaohui Zhai, Dan Cao, Shaoqing Gong

**Affiliations:** 1 School of Public Policy and Administration, Xi'an Jiaotong University, No. 28 Xianning West Road, Xi'an, 710049, China, 86 18629510661; 2 School of Public Health, Health Science Center, Xi'an Jiaotong University, Xi'an, China; 3 School of Public Administration, Xi’an University of Architecture and Technology, Xi’an, China; 4 Luohe Medical College, Luohe, China

**Keywords:** digital health technology, physician adoption, latent profile analysis, heterogeneous adoption profiles, implementation strategies

## Abstract

**Background:**

Digital health technologies (DHTs) are transforming global health care delivery, yet physician adoption remains highly variable and influenced by a complex interplay of individual, institutional, and technological factors. In China, despite national initiatives such as the “Healthy China 2030” strategy promoting DHT integration, understanding physicians' heterogeneous perceptions is essential for effective implementation.

**Objective:**

This study aimed to identify distinct latent profiles of Chinese physicians based on their perceptions of DHT benefits, barriers, and behavioral intention, and to examine the demographic and occupational factors associated with profile membership.

**Methods:**

A cross-sectional survey was conducted among 4851 physicians (female, n=2994, 60.69% ; mean age 38.4, SD 8.7 years; 51.58% (n=2502) with more than 10 y working experience) from 46 hospitals in Shaanxi Province, China, between October and December 2023. Assessment included nine indicators across three domains: Perceived Benefits (4 items), Adoption Barriers (4 items), and Behavioral Intention (1 item). Latent profile analysis was used to identify distinct subgroups of physicians based on their response patterns. Multinomial logistic regression examined predictors of profile membership, and results were reported as odds ratios (ORs) with 95% CIs.

**Results:**

The latent profile analysis identified 5 distinct profiles: Reform-Adaptable (n=516, 10.64%; 95% CI 9.76%-11.52%), Negative (n=1003, 20.68%; 95% CI 19.50%-21.86%), Neutral (n=2276, 46.92%; 95% CI 45.50%-48.34%), Reform-Conservative (n=545, 11.23%; 95% CI 10.33%-12.13%), and Positive (n=511, 10.53%; 95% CI 9.66%-11.40%). Significant intergroup differences were observed in demographic and occupational characteristics. For instance, compared with the Negative profile, male physicians were less likely to belong to the Neutral (OR 0.76, 95% CI 0.64‐0.90; *P*=.001) and Reform-Conservative (OR 0.67, 95% CI 0.54‐0.84; *P*=.001) profiles. Compared to the Neutral profile, physicians with a master’s degree or above were less likely to be in the Reform-Conservative profile (OR 0.75, 95% CI 0.59‐0.96; *P*=.052). Those working in tertiary hospitals were less likely to belong to the Positive group (OR range 0.56‐0.66, *P*=.001). High-income physicians were more likely to be in the Reform-Conservative group (OR range 1.83‐2.38, *P*=.001). In addition, higher occupational stress was associated with a greater likelihood of Positive profile membership (OR range 1.12‐1.26, *P*=.001), while better work satisfaction predicted higher odds of Positive profile membership (OR range 1.04‐1.16, *P*=.001).

**Conclusions:**

This study introduces a novel, person-centered approach by identifying five distinct perceptual typologies among physicians, moving beyond traditional variable-centered analyses. This typology provides an evidence-based foundation for tailored interventions. For instance, the Reform-Adaptable group may need barrier reduction, while the Reform-Conservative group may require clearer value demonstrations. This nuanced understanding can help healthcare systems enhance the impact and scalability of digital health technologies in real-world clinical practice.

## Introduction

### Background

The global proliferation of digital health technologies (DHTs), ranging from telemedicine to artificial intelligence (AI)-driven diagnostics, has reshaped health care delivery [1]. These innovations offer significant potential to address global health system challenges by improving service coverage, health care efficiency, and the quality of health care practices and services [2,3]. Within this global context, China has actively promoted DHT adoption through its “Healthy China 2030” initiative, which specifically aims to develop interoperable health data platforms, facilitate cross-sector medical collaboration, and reduce urban-rural health care disparities [4]. However, despite these advancements, the adoption and usage of DHTs among physicians remain uneven, influenced by a complex interplay of factors [5]. At the organizational level, existing research has established that institutional support systems (eg, training and technical assistance) and conducive regulatory environments are critical contextual facilitators of DHT adoption [6]. Conversely, growing evidence underscores that individual cognitive factors may be even more pivotal in shaping physicians’ decisions—such as perceived usefulness and ease of use, self-efficacy in using DHTs, and deeply held mental models about clinical workflows. Nevertheless, the field lacks robust evidence to explain how these cognitive mechanisms account for the substantial variations observed in physicians’ DHT adoption patterns, particularly across different clinical contexts and implementation stages [5,7]. These variations appear to originate from both methodological differences in how studies measure technology acceptance and unaddressed heterogeneity among physician populations, particularly across different medical specialties and practice settings. This study addresses this gap by applying latent profile analysis (LPA) to identify distinct subgroups of physicians based on their personal evaluations of DHT adoption. Given the central role of physicians in the digital transformation of health care, understanding their perspectives is essential for ensuring the successful implementation and widespread adoption of these technologies.

### DHT Adoption Landscape

The term “digital health,” which evolved from “eHealth,” refers to the application of information and communication technologies to support health care and health-related fields. More recently, “digital health” has been introduced as a broader concept encompassing eHealth (including mobile health) and emerging fields such as the application of advanced computing sciences in data, genomics, and AI [2]. The adoption of DHT services to support patient care has grown significantly in health care institutions worldwide. Driven by the increasing prevalence of mobile phones and the widespread availability of preventive health and fitness applications, DHT and eHealth are playing an increasingly important role in enhancing medical workflows [6]. However, while digital health solutions are increasingly popular with the public, implementation faces hurdles in clinical settings. A central challenge is the lack of systematic frameworks to rigorously evaluate both benefits and risks. This evaluation gap contributes to professional hesitancy among health care providers and institutions, limiting user engagement and contributing to differences in technology uptake across care settings [5]. Recent literature confirms that DHT adoption rates exhibit significant variation across different service types, clinical specialties, and patient subgroups [8]. Moreover, the underusage of DHT poses considerable difficulties for modern health care systems. Hospitals experience decreased operational efficiency, reduced care quality, and financial strain due to factors such as patient attrition and restricted insurance reimbursements [2]. In turn, patients’ limited access to DHT may lead to suboptimal care, including extended waiting times, which further widens existing health disparities [9]. Therefore, effectively addressing these DHT adoption challenges is essential for promoting sustainable, equitable, and patient-centered health care delivery in the future.

### Determinants of Uneven DHT Adoption

The heterogeneous adoption patterns of DHTs stem from a dynamic interaction between enabling factors and systemic barriers. When DHTs demonstrate measurable clinical effectiveness, health care providers are more likely to recognize their potential for enhancing work efficiency and patient outcomes, thereby developing favorable attitudes toward technology adoption. This positive perception creates a virtuous cycle that may ultimately improve clinical performance [10]. Conversely, inadequate integration of DHTs with existing clinical workflows often generates resistance among health care professionals, potentially undermining implementation efforts [11].

Current evidence frames DHT adoption through a tripartite model integrating: (1) individual factors (eg, perceived utility vs digital literacy gaps); (2) organizational and environmental factors (eg, supportive policies vs financial constraints); and (3) technological factors (eg, interoperability vs security risks) [6]. Among physicians, adoption barriers are particularly multifaceted, spanning cognitive (eg, technophobia), attitudinal (eg, skepticism toward clinical efficacy), and experiential domains (eg, limited previous exposure). Resistance often stems from perceived workflow disruptions, eroded patient-provider dynamics, or mismatches between technology design and clinical needs. Conversely, demonstrable efficiency gains, user-friendly interfaces, and alignment with professional norms foster acceptance. Critically, adoption patterns reflect an interplay of these dimensions; for instance, even robust technology may fail if organizational support (eg, training) is lacking [5,12]. Tailored strategies addressing domain-specific barriers (eg, pilot programs for technophobic clinicians and interoperable tools for fragmented systems) are essential to bridge gaps between policy goals and real-world implementation [6].

The Unified Theory of Acceptance and Use of Technology 2 (UTAUT 2) has been effectively applied across international contexts, including Germany and the United States, to examine DHT adoption. Studies based on this framework, which often incorporate constructs such as perceived security and relative advantage and use age-stratified sampling, consistently identify performance expectancy and hedonic motivation as key drivers of usage intention. These studies also highlight security concerns as a major barrier [13]. Further research on German mobile health apps revealed the predominant influence of hedonic motivation over utilitarian factors, with contextual variations observed between lifestyle and therapeutic apps [14]. Collectively, these findings underscore the adaptability of UTAUT 2 across diverse health care technologies and cultural settings, particularly when incorporating domain-specific variables. However, research based on UTAUT 2 remains largely confined to conventional methods such as subgroup analyses and clustering approaches, which rely on variable-centered techniques such as moderation analysis or predefined demographic comparisons. These methodological constraints may limit the ability to capture clinically meaningful, person-oriented adoption profiles [13]. Realizing the full generalizability of DHT adoption models requires not only careful consideration of user and provider heterogeneity, along with further validation across diverse populations, but also the adoption of more nuanced, person-centered analytical frameworks. A comprehensive understanding of physicians’ adoption behaviors demands a multidimensional perspective that simultaneously assesses perceptions of utility, risks, barriers, and usage intentions, ultimately moving beyond structural models toward person-centered approaches.

Despite physicians’ pivotal role as clinical decision-makers and primary end users of DHTs, current research predominantly centers on citizen [14] and patient perspectives [6,15], or on technical feasibility [16], leaving a significant gap regarding health care professionals’ perceptions and experiences. Few studies have specifically targeted the evaluation of the creation, implementation, long-term use, and self-reported barriers and facilitators to DHT use by health care professionals [5]. Moreover, the majority of existing studies, including those using established theoretical frameworks such as the technology acceptance model [17] and the UTAUT model [18], rely predominantly on variable-centered approaches. These approaches focus on the relationship between DHT or eHealth service implementation and various factors across the overall sample. From this perspective, most previous studies—including those using UTAUT 2—focus on aggregate relationships and isolated moderators, thereby overlooking systematic heterogeneity within physician populations. Such constraints ultimately diminish their capacity to explain actual usage patterns within complex health care environments. More critically, such variable-centered methods inherently assume population homogeneity and thus obscure meaningful heterogeneity across distinct user subgroups, leading to an inadequate characterization of clinically relevant adoption patterns and context-specific barriers. This gap is especially pronounced in the Chinese context, where rapid, policy-driven digital health transformation may have generated unique adoption profiles not captured by conventional approaches.

### Study Rationale and Objectives

To address these limitations, this study introduces LPA as a novel, person-centered methodological framework for investigating physician adoption of DHTs. LPA is a probabilistic modeling technique that identifies naturally occurring subgroups within multidimensional data based on shared response patterns [19]. This method is particularly valuable for capturing heterogeneity and identifying nuanced profiles of technology acceptance that remain concealed in variable-level analyses [20,21]. In contrast to previous variable-centered studies, LPA enables (1) the identification of clinically meaningful subgroups characterized by distinct configurations of perceptions across benefits, barriers, and behavioral intentions; (2) the examination of multilevel predictors of subgroup membership; and (3) the development of tailored implementation strategies aligned with the specific needs of different physician populations. Given physicians’ pivotal role in health care’s digital transformation, these insights are critical for developing targeted interventions that move beyond one-size-fits-all adoption strategies to account for the nuanced needs and perceptions of different clinician subgroups [22].

Therefore, this study is designed to achieve 2 key objectives. First, it aims to classify Chinese physicians’ DHT preferences using LPA to identify heterogeneous subgroups based on a 3D evaluation framework. Second, it seeks to investigate how demographic and occupational factors correlate with profile membership. By transcending aggregate-level insights, this approach offers a more nuanced and clinically relevant understanding of DHT adoption behaviors. As DHTs become increasingly prevalent, the findings are poised to inform tailored interventions that address implementation barriers, especially among hesitant health care professionals. Furthermore, this research provides actionable recommendations for policymakers, health authorities, medical institutions, and insurers to support the design of context-sensitive DHT adoption strategies that enhance physician engagement and ultimately improve health care delivery.

## Methods

### Study Design and Data Sources

With the approval of the Shaanxi Provincial Health Commission and authorization from the Xi’an Municipal Health Commission, we undertook a cross-sectional investigation across health care facilities in Xi’an, Shaanxi Province, China. This investigation, conducted from October 18 to December 23, 2023, was a crucial part of the “2023 Healthcare Worker Survey” and the broader 7th Xi’an Health Services Survey. The survey aimed to evaluate medical staff’s practice status, working conditions, and health to inform local health policy and management. It has also been used in previous studies on health care professionals’ well-being and occupational challenges [23]. This study used a cross-sectional survey design, conducted in accordance with the STROBE (Strengthening the Reporting of Observational Studies in Epidemiology) guidelines ( [24]).

We used random cluster sampling to select 46 hospitals (26 Level-II and 20 Level-III) from municipal and county-level medical institutions in Xi’an. Eligible participants included licensed physicians (including therapists and clinical practitioners) with full-time or contractual employment status in either public or private hospitals. To ensure sample homogeneity and mitigate potential selection bias, we restricted our sample to physicians affiliated with institutions that had formally implemented DHT programs. This inclusion criterion accounted for self-selection bias, given that physicians who had adopted DHT voluntarily before institutional rollout might have exhibited systematically more favorable attitudes toward DHT than the broader physician population (detailed information on the data resources is provided in Multimedia Appendix 1).

To ensure data quality, we conducted a pilot test with 814 health care workers (achieving 93.5% compliance) and trained liaison officers from 33 city-level hospitals and 9 county-level government departments on survey protocols, quality control, and tool usage. We implemented a range of data quality control measures, including consistency checks (eg, control questions 12 and 55), logic verification (eg, years of service), outlier detection (eg, age range), and completion time analysis (requiring >3 minutes for >90% completion). From an initial 8617 responses, 3766 were excluded due to incomplete data (n=283), invalid entries (n=97), excessively short completion times (n=46), or employment at institutions where the relevant DHT was not implemented or its status was unknown (n=3431). The remaining 4851 responses were included in the final analysis (detailed Missing Completely At Random test results are provided in Section 2, Multimedia Appendix 1).

### Demographic and Occupational Characteristics of Participants

Drawing on previous literature regarding barriers and facilitators of DHT adoption, which highlights the association between certain sociodemographic and occupational characteristics (eg, age, gender, professional title, and years of experience) [5] and DHT adoption, we included similar indicators in our analysis to examine their association with profile membership. Specifically, the sociodemographic and occupational factors assessed in this study comprised: (1) sociodemographic factors such as gender, age, educational attainment (Bachelor’s, Master’s, or PhD), annual income level (stratified by tertiles), and self-rated health status (5-point Likert scale: 1=very poor to 5=excellent); and (2) occupational variables such as hospital grade (Level-II [secondary] vs Level-III [tertiary], professional title [resident, attending, or chief physician]), years of clinical experience, weekly working hours, monthly night shift frequency, as well as psychosocial measures including work satisfaction (assessed using a 10-item scale), occupational stress (4-item scale), and doctor-patient relationship quality (3-item scale).

### Doctor-Patient Relationship Quality Scale

Physicians’ perceptions of the doctor-patient relationship were measured using the DPRQ-3 (Doctor-Patient Relationship Questionnaire-3), a simple and easy-to-use questionnaire designed for assessing the doctor-patient relationship in medical settings, and served as the primary independent variable [25]. This 3-item scale includes questions such as: “How do you feel patients respect the doctor?”, “To what extent do you believe society respects the doctor profession?”, and “What do you think of the current doctor-patient relationship?”. Participants answered each item using a 5-point Likert scale (1=very disrespectful or very bad to 5=very respectful or very good). In this paper, the Cronbach α coefficient of this scale was 0.82.

### Occupational Stress Scale

In this study, occupational stress is defined as the stressful aspects of clinical work encountered by physicians in their professional environment. The occupational stress scale was adapted from existing instruments to measure the psychological distress perceived by medical staff while performing their duties [26,27]. Participants responded to 4 items on a 6-point Likert scale ranging from 1 (strongly disagree) to 6 (strongly agree). These items included: “Overall, I feel great pressure at work,” “I feel a high level of tension at work,” “I’m having trouble sleeping because of work,” and “I’m nervous about going to work.” Selected items capture core dimensions of nursing stress (global pressure, tension, sleep disturbance, and work avoidance), aligning with Lazarus’s transactional stress model [28]. This scale is a validated tool that has been extensively used as a measure of job pressure and psychological distress in both medical staff and general occupational research, thus demonstrating its applicability to this study [25]. The total scores ranged from 4 to 24 and demonstrated high internal consistency (Cronbach *α*=0.94; composite reliability=0.88).

### Work Satisfaction Scale

Work satisfaction was measured using a 10-item scale assessing several dimensions: overall job satisfaction, satisfaction with colleagues, expected income, leadership, working facilities, promotion prospects, internal management, welfare benefits, training opportunities, and opportunity for skill use [5]. Participants rated each item on a 6-point Likert scale ranging from “1=very dissatisfied to 6=very satisfied,” resulting in a total score from 10 to 60. The scale exhibited excellent internal consistency (Cronbach *α*=0.95). The full details of the scale are provided in Part B of Multimedia Appendix 2.

### Digital Health Care Technology Adoption Scale

Current literature indicates that both the general public and health care professionals widely recognize the significant potential benefits and barriers associated with DHTs [6,7,29] or eHealth services [30]. With the aim of thoroughly investigating practicing physicians’ perspectives and preferences related to the implementation of DHTs, we developed a 14-item DHT adoption scale comprising 3 dimensions, based on a comprehensive literature review [5,29]. The scale development process, including expert validation procedures and pilot testing protocols, is provided in detail in Section 2, Multimedia Appendix 3. Specifically, the selection of the 3 core dimensions—Perceived Benefits, Adoption Barriers, and Behavioral Intention—was guided by established technology adoption theories, notably the technology acceptance model and the UTAUT theories, which posit that behavioral intention is determined by a trade-off between perceived benefits (eg, usefulness) and perceived costs or barriers (eg, ease of use and risks) [13]. Also, recognizing that personal preference does not always translate into actual use, we incorporated a third dimension, Behavioral Intention, to capture a more behavioral measure of overall adoption willingness. This tripartite structure allows for a more comprehensive assessment that spans attitudinal, perceptual, and behavioral aspects of adoption.

Within the Perceived Benefits domain, which consists of 8 items, 4 specific indicators were identified as the most frequently cited drivers of DHT adoption in systematic reviews and physician surveys. These indicators include (1) improved diagnostic and treatment quality, (2) enhanced patient trust and satisfaction, (3) error rate reduction, and (4) increased income (driven by improved diagnostic and treatment efficiency) [5,29]. From the physician’s perspective, these represent core utilitarian, relational, and practical incentives. Similarly, the Adoption Barriers domain contains 5 items, with 4 key indicators consistently highlighted in previous literature as the most prevalent and impactful obstacles. These indicators comprise (1) technical barriers, (2) cybersecurity risks, (3) workload increase, and (4) patient experience reduction [6,29], reflecting central concerns regarding feasibility, security, and clinical workflow. The third dimension, Behavioral Intention, was assessed using a single-item scale designed to measure overall willingness to adopt. This provides a pragmatic measure of behavioral outcomes, complementing the multidimensional perceptual factors. Taken together, this framework ensures the scale captures both the complexity of DHT adoption decisions and a concrete behavioral intention.

All items were rated on a 5-point Likert scale, with each indicator score standardized to a range of 1 to 5. Higher scores in the Perceived Benefits domain indicated that participants recognized greater potential benefits of DHTs, whereas lower scores in the Adoption Barriers domain suggested that participants perceived higher potential costs and risks associated with DHT implementation. Correspondingly, higher scores in the Behavioral Intention domain demonstrated increased likelihood of both initial adoption and sustained usage of DHTs. The scope of DHTs considered in this study and the specific items included in the DHT scale are provided in Part A of Multimedia Appendix 2. This scale demonstrated high internal consistency, with a Cronbach α of 0.88. Detailed information regarding the validity of the scale is provided in Table S5 of Multimedia Appendix 3.

### Data Analysis

Descriptive statistics and bivariate correlations were analyzed using Stata 17 (StataCorp LLC). Mplus version 8.3 (Muthén & Muthén) software was used to conduct the LPA and identify the DHT subgroups based on 9 domains (4 benefit domains, 4 barrier domains, and 1 objective domain). We assessed model fit using a comprehensive set of indices [31], including the Akaike information criterion (AIC), Bayesian information criterion (BIC), adjusted BIC (aBIC), entropy, the Lo-Mendell-Rubin likelihood ratio test, and the bootstrap likelihood ratio test (BLRT). Lower values of AIC, BIC, and aBIC indicated better model fit [10]. The Lo-Mendell-Rubin likelihood ratio test and BLRT were used to compare improvements in model fit between adjacent models, with a significant *P* value (*P*<.05) suggesting that the class-k model provided a better fit than the class k-1 model. Entropy values, ranging from 0 to 1, were used to evaluate classification quality, with values closer to 1 indicating clearer class separation. In addition, the average posterior probability of class membership was examined, with values ≧0.80 indicating good discriminability. To ensure the validity of the results, each class was required to comprise more than 5% of the total sample [19]. The uncertainty in the estimated latent profile proportions was quantified using 95% CIs, constructed via a nonparametric bootstrap approach with 1000 replications. This method is robust and does not rely on distributional assumptions, making it particularly suitable for latent variable models.

Next, we performed ANOVA to compare DHT subscale scores across the 5 latent classes. Between-group differences in demographic, health, and occupational characteristics across DHT subtypes were assessed using *χ*^2^ tests (for categorical variables) and ANOVA (for continuous variables). To examine the relationships between the identified DHT profiles and key variables, we performed multivariate multinomial logistic regression analyses. Multicollinearity was assessed using variance inflation factor analysis (Table S4 in Multimedia Appendix 3). These models assessed the associations between DHT profiles and various predictors, with statistical significance determined at *P*<.05 (2-tailed).

### Ethical Considerations

This study collected solely demographic and professional information, excluding any sensitive or personally identifiable biological data. The study protocol was approved by the Biomedical Ethics Committee of Xi’an Jiaotong University (approval no XJTUAE-2647). Electronic informed consent was obtained from all participants, and institutional authorization was granted by the Xi’an Municipal Health Commission. For the secondary analysis of the research data, we confirmed that the original ethical approval and consent procedures for the “2023 Healthcare Worker Survey” permitted the reuse of data for public health and policy studies without additional participant consent.

In this study, we prioritized the privacy and confidentiality of participants. The survey was designed to collect only nonsensitive information without any personally identifiable data. All data were deidentified at the time of collection, and analyses were conducted on aggregated datasets to prevent reidentification. Participants were not offered any form of compensation, as the survey was part of routine institutional activities. No images or multimedia materials that could lead to the identification of any individual are included in the paper or supplementary files.

## Results

### Descriptive Statistics and Correlations

A total of 4851 Chinese registered doctors from 46 health care facilities (including 26 Level-II hospitals and 20 Level-III hospitals) in Xi’an were analyzed in this study. The mean age was 38.37 (SD 8.67) years, with a range of 20 to 80 years. Among the participants, 2944 (60.69%) were female, and 1907 (39.31%) were male. In terms of education, 56.17% (2725/4851) held graduate degrees (master’s or doctoral degrees), while 43.83% (2126/4851) had a bachelor’s degree or below.

Among the 9 items in the DHT perception scale, the diagnosis and treatment quality indicator had the highest mean score of 3.98 (SD 0.78) in the benefit domain, while the income increase indicator had the lowest mean score of 3.08 (SD 1.01). In the barrier domain, the patient experience reduction indicator had the highest mean score of 3.80 (SD 0.96), whereas the workload increase indicator had the lowest mean score of 3.59 (SD 0.98). The mean score for the overall willingness indicator was 3.69 (SD 0.89). In terms of job-related scales, the mean scores for work satisfaction, occupational stress, and doctor-patient relationship perception were 44.30 (SD 9.69), 16.22 (SD 4.85), and 7.85 (SD 2.08), respectively. The bivariate correlations among the study variables are provided in Table S1 of Multimedia Appendix 3. All indicators of DHT were moderately correlated; furthermore, compared to correlation analysis, LPA offers a more detailed characterization of Chinese doctors’ diverse perspectives on DHT.

### Detecting Latent Profiles

The model fit statistics for the 1‐6 latent profile models are provided in Table 1. With an increase in the number of latent profiles, the AIC, BIC, and aBIC gradually decreased, and the BLRT showed significant results in comparisons between all models with k and k–1 classes. Although the class-6 model demonstrated the best fit based on AIC, BIC, aBIC, and entropy, the first group in this model included only 77 participants (1.6% of the total sample), leading to the rejection of the class-6 model. Compared to the class-4 model, the class-5 model identified a new category with a distinct DHT-related response probability pattern. Based on its optimal balance of model fit and interpretability, the class-5 model was selected as the final solution. This model showed the highest classification accuracy among comparable models, with an entropy value of 0.883, indicating well-separated and mutually exclusive profiles. This finding is further supported by the high average posterior class probabilities provided in Table S3 in Multimedia Appendix 3.

**Table 1.** Model fit indices for the compared latent profile analysis models evaluating digital health technology adoption among physicians in China (cross-sectional survey, 2023; N=4851).

Model	AIC^a^	BIC^b^	aBIC^c^	pLMR^d^	pBLRT^e^	Entropy	Group size for each profile					
							1	2	3	4	5	6
Class-1	113430.03	113546.79	113489.59	^f^—	—^f^	—	4851	—	—	—	—	—
Class-2	107352.93	107534.56	107445.59	<.001	<.001	0.760	2292	2559	—	—	—	—
Class-3	102959.52	103206.02	103085.26	<.001	<.001	0.830	2326	617	1908	—	—	—
Class-4	99087.54	99398.91	99246.38	<.001	<.001	0.882	1120	584	2485	562	—	—
Class-5	96769.86	97146.10	96961.80	<.001	<.001	0.883	516	1003	2276	545	511	—
Class-6	95262.60	95703.71	95487.65	<.001	<.001	0.889	528	77	1149	2082	498	517

a AIC: Akaike information criterion.

b BIC: Bayesian information criterion.

c ABIC: adjusted BIC.

d pLMR*: P* value for LoMendell-Rubin adjusted likelihood ratio test for K versus K–1 profiles.

e pBLRT*: P* value for bootstrapped likelihood ratio test.

f Not applicable.

**Figure 1.**
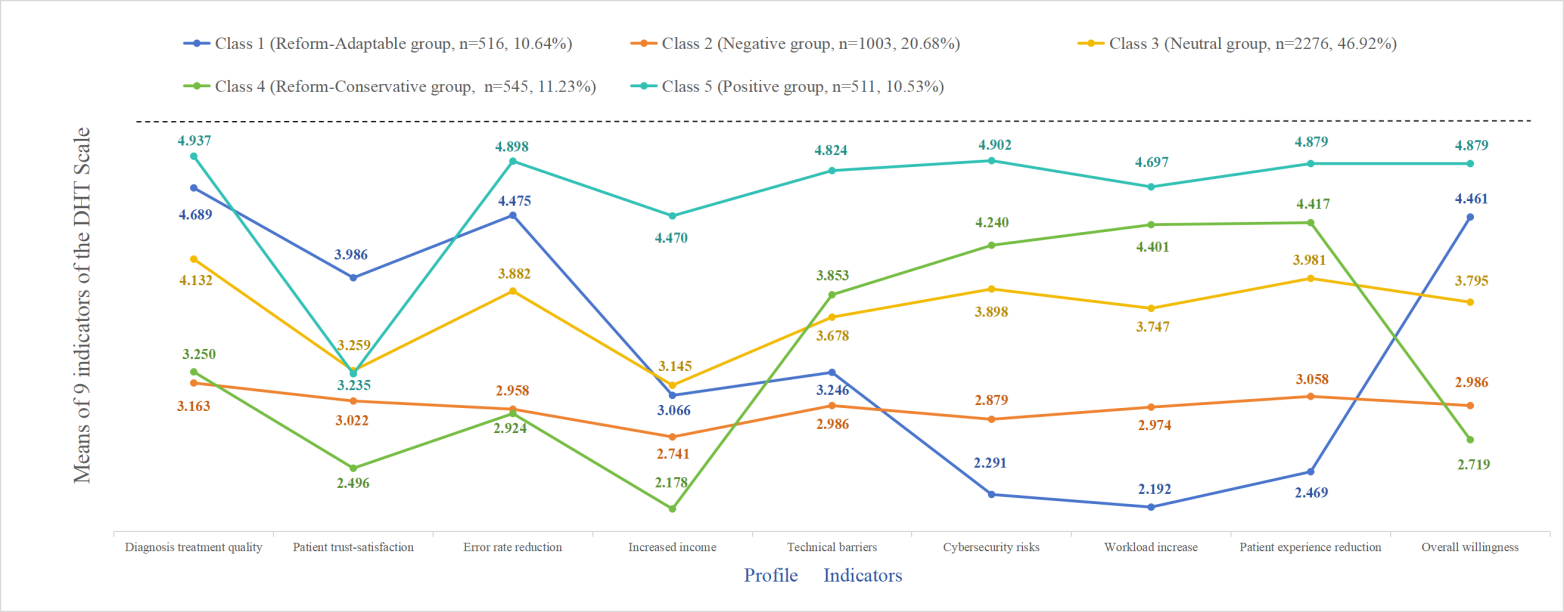
Characteristics of the 5 digital health technology (DHT) adoption profiles identified by latent profile analysis among hospital-based physicians in China (cross-sectional survey, 2023; N=4851), based on patterns of Perceived Benefits, Adoption Barriers, and Behavioral Intention.

The latent profile memberships showed significant differences in the means of the 8 indicator variables (as provided in Table S2 in Multimedia Appendix 3), and their characteristics are summarized in Figure 1. The LPA was conducted to identify physician subgroups based on their standardized responses (on a 1–5 scale) across 3 key domains: Perceived Benefits, Adoption Barriers, and Behavioral Intention. The Perceived Benefits domain encompassed four indicators: (1) improved diagnostic and treatment quality, (2) enhanced patient trust and satisfaction, (3) error rate reduction, and (4) increased income. The Adoption Barriers domain included: (1) technical barriers, (2) cybersecurity risks, (3) workload increase, and (4) patient experience reduction. The Behavioral Intention domain measured the overall willingness to adopt. In the resulting profiles (Figure 1), higher scores in Perceived Benefits and Behavioral Intention indicate more positive perceptions and a greater likelihood of adoption, respectively. Conversely, higher scores in Adoption Barriers signify that physicians perceived these obstacles as more severe. The ANOVA and Bonferroni post hoc tests indicated that DHT subscale scores differed in all 5 classes (*P*<.001), with the “Error Rate Reduction” variable exhibiting the largest effect size (η^2^=0.627). In Figure 1, Class 1 (n=516, 10.64% of the sample; 95% CI 9.76%-11.52%) demonstrated a distinctive pattern characterized by high perceived benefits, high perceived barriers, yet positive overall willingness toward DHTs. This profile represents physicians who recognize both notable advantages and substantial risks of digital health tools, but tend to maintain a generally positive willingness to adopt and use these technologies. Their pattern could suggest a risk-aware yet largely optimistic approach to digital transformation, potentially serving as engaged evaluators who might help optimize DHT implementation while acknowledging its challenges. This unique profile was therefore classified as the “Reform-Adaptable” group. Class 2 (n=1003, 20.68% of the sample, 95% CI 19.50%-21.86%) exhibited consistently low scores across all dimensions, suggesting generally skeptical attitudes toward DHTs. This profile appears to reflect physicians who perceive relatively minimal benefits while emphasizing substantial barriers, resulting in largely negative adoption intentions. Their resistance seems rooted in both practical concerns about implementation challenges and some fundamental doubts about the value of DHTs. This group was designated the “Negative” group. Class 3 (n=2276, 46.92% of the sample; 95% CI 45.50%-48.34%) was characterized by moderate scores near the average on all subscales. We interpret this pattern as representing physicians who acknowledge both the advantages and limitations of DHTs without a firm stance. This neutral position likely entails a “wait-and-see” approach, where adoption is contingent on contextual factors such as organizational support and peer behavior. Based on this rationale, we identified this group as the “Neutral” profile. Class 4 (n=545, 11.23% of the sample; 95% CI 10.33%-12.13%) presented a profile of low perceived benefits, low perceived barriers, and cautious overall willingness. These physicians appear to perceive limited advantages from DHTs while also minimizing implementation risks, resulting in generally low adoption intentions that seem based more on skepticism about the fundamental value proposition of DHTs rather than specific implementation concerns. This group was therefore labeled the “Reform-Conservative” group. Class 5 (n=511, 10.53% of the sample; 95% CI 9.66%-11.40%) displayed uniformly high scores across all subscales, implying favorable dispositions toward DHTs. This profile may represent physicians who recognize strong benefits, tend to minimize perceived barriers, and demonstrate relatively high adoption willingness. Their pattern suggests generally positive acceptance of digital transformation and potential leadership roles in promoting DHT implementation within their institutions. Consequently, this group was classified as the “Positive” group.

### Comparison of Demographic and DHT Scales in Each Latent Profile

Table 2 outlines the comparison of demographic and job-related variables across different latent profiles. Significant differences were observed among the 5 DHT classes for variables such as gender, education background, income level, professional and technical title, working hours per week, years of health care work experience, self-rated health, work satisfaction, doctor-patient relationship perception, and occupational stress (all *P*<.05). However, no significant differences were found for age and night shift status across the 5 DHT profiles.

**Table 2.** Association between identified digital health technology adoption profiles and demographic and occupational characteristics among physicians in China (cross-sectional survey, 2023; N=4851).

Variable	Overall (N=4851)	Class 1^a^ (n=516 10.64%)	Class 2^b^ (n=1003 20.68%)	Class 3^c^ (n=2276 46.92%)	Class 4^d^ (n=545 11.23%)	Class 5^e^ (n=511 10.53%)	^g^Test statistic, Chi-square (df)/ *F* (df1, df2)
Continuous variables, mean (SD)							
Age (years)	38.37 (8.67)	38.05 (8.77)	39.28 (8.92)	37.76 (8.46)	39.84 (8.55)	38.03 (8.84)	4.92 (4,4846)
Self-rated health status (range 1‐5)	3.30 (0.80)	3.64 (0.83)	3.16 (0.76)	3.31 (0.77)	3.04 (0.73)	3.41 (0.88)	27.58 (4,4846)^f^
Work Satisfaction Scale (range 10‐60)	44.30 (9.69)	50.13 (8.87)	41.03 (8.80)	44.23 (8.45)	38.33 (9.65)	51.49 (9.92)	32.27 (4,4846)^f^
Doctor-Patient Relationship Quality Scale (range 3‐15)	7.85 (2.08)	6.72 (2.04)	8.30 (1.95)	7.79 (1.84)	8.96 (2.01)	7.19 (2.55)	100.42 (4,4846)^f^
Occupational Stress Scale (range 4‐24)	16.22 (4.85)	12.83 (5.57)	16.06 (4.10)	16.17 (4.45)	17.45 (4.05)	18.82 (5.75)	148.27 (4,4846)^f^
Categorical variable, n (%)							
Gender							27.22 (4)^h^^,^^f^
Female	2994 (60.69)	304 (58.91)	576 (57.43)	1454 (63.88)	338 (62.02)	272 (53.23)	
Male	1907 (39.31)	212 (41.09)	427 (42.57)	822 (36.12)	207 (37.98)	239 (46.77)	
Education background							15.50 (4)^f^^,^^h^
Bachelor’s degree and below	2126 (43.83)	205 (39.73)	447 (44.57)	976 (42.88)	237 (43.49)	261 (51.08)	
Master’s degree and above	2725 (56.17)	311 (60.27)	556 (55.43)	1300 (57.12)	308 (56.51)	250 (48.92)	
Hospital grade							38.32 (4)^f^^,^^h^
Level-II	1174 (24.2)	99 (19.19)	231 (23.03)	573 (25.18)	103 (18.90)	168 (32.88)	
Level-III	3677 (75.8)	417 (80.81)	772 (76.97)	1703 (74.82)	442 (81.10)	343 (67.12)	
Professional and technical title							44.96 (8)^f^^,^^h^
Resident physician	1231 (25.38)	143 (27.71)	224 (22.33)	617 (27.11)	101 (18.53)	146 (28.57)	
Attending physician	1938 (39.95)	207 (40.12)	390 (38.88)	930 (40.86)	207 (37.98)	204 (39.92)	
Chief physician	1682 (34.67)	166 (32.17)	389 (38.78)	729 (32.03)	237 (43.49)	161 (31.51)	
Annual income level							51.52 (8)^f^^,^^h^
Low	1671 (34.45)	170 (32.95)	360 (35.89)	789 (34.67)	141 (25.87)	211 (41.29)	
Middle	1765 (36.38)	190 (36.82)	364 (36.29)	858 (37.70)	184 (33.76)	169 (33.07)	
High	1415 (29.17)	156 (30.23)	279 (27.82)	629 (27.64)	220 (40.37)	131 (25.64)	
Working hours							34.53 (4)^f^^,^^h^
≤48 h/wk	2612 (53.84)	311 (60.27)	517 (51.55)	1260 (55.36)	240 (44.04)	284 (55.58)	
>48 h/wk	2239 (46.16)	205 (39.73)	486 (48.45)	1016 (44.64)	305 (55.96)	227 (44.42)	
Night shifts							1.58 (4)^h^
≤4 nights/time per month	2219 (45.74)	246 (47.67)	455 (45.36)	1037 (45.56)	255 (46.79)	226 (44.23)	
>4 nights/time per month	2632 (54.26)	270 (52.33)	548 (54.64)	1239 (54.44)	290 (53.21)	285 (55.77)	
Health care working experience							22.57 (4)^f^^,^^h^
≤10 years	2349 (48.42)	263 (50.97)	448 (44.67)	1156 (50.79)	227 (41.65)	255 (49.90)	
>10 years	2502 (51.58)	253 (49.03)	555 (55.33)	1120 (49.21)	318 (58.35)	256 (50.10)	

a Class 1: Reform-Adaptable group.

b Class 2: Negative group.

c Class 3: Neutral group.

d Class 4: Reform-Conservative group.

e Class 5: Positive group.

f *P*<.001*.*

g ANOVA *F* tests are used for continuous variables; *F* (df1, df2).

h Chi-square tests (*χ²* tests) are used for categorical variables; Chi-square (df).

As shown in Table 2, the Positive group (Class 5) demonstrated significantly higher proportions of participants affiliated with Level-II hospitals (*χ*^2^_4_=38.32; *P*<.001), holding resident physician titles (*χ*^*2*^_8_=44.96; *P*<.001), and possessing bachelor’s degrees (*χ*^2^_4_=15.50; *P*<.001) compared with other groups. Notably, this group also reported the highest mean scores in both work satisfaction (mean 51.49, SD 9.92) and occupational stress (mean 18.82, SD 5.75).

### Multivariate Multinomial Regression Results

Tables3  4 show the associations between key *p*redictors and latent profile membership, using the subsequent class in each column as the reference. Male physicians were less likely to belong to the Neutral (Class 3) and Reform-Conservative (Class 4) groups compared with both the Reform-Adaptable (Class 1) and Negative (Class 2) groups (all odds ratios [ORs] <1), but more likely to belong to the Positive group (Class 5) than to Class 4 (OR 1.39, 95% CI: 1.05-1.84; *P*=.02). Those with a master’s degree or higher were less likely to be in Class 4 than Class 3 (OR 0.75, 95% CI 0.59‐0.96; *P*=.02). When using Class 2 as the reference, better self-rated health was significantly associated with higher odds of belonging to Class 1 (OR 1.21, 95% CI 1.03‐1.42; *P*=.02), Class 3 (OR 1.20, 95% CI 1.07‐1.34; *P*=.001), and Class 5 (OR 1.32, 95% CI 1.12‐1.55; *P*=.001). These graded associations indicate that gender, education, and self-rated health are important differentiating factors across distinct DHT perception profiles. However, contrary to expectations derived from existing literature, our findings revealed that age, professional title, and years of work experience did not significantly predict DHT adoption profile membership among physicians in the Chinese sample (all *P*>.05), suggesting important contextual differences in the determinants of DHT adoption.

**Table 3.** Multinomial logistic regression results (Part A) examining the demographic and occupational predictors of membership in the 5 digital health technology adoption profiles among Chinese physicians (cross-sectional survey, 2023; N=4851).

Variable	Class 5^e^ vs Class 1^a^, ^f^OR (95% CI)	Class 5 vs Class 2^b^, OR (95% CI)	Class 5 vs Class 3^c^, OR (95% CI)	Class 5 vs Class 4^d^, OR (95% CI)	Class 2 vs Class 1, OR (95% CI)
Age (years)	0.99 (0.96‐1.02)	0.98 (0.95‐1.00)	1.00 (0.98‐1.03)	0.99 (0.96‐1.01)	1.01 (0.98‐1.04)
Gender (ref: female)					
Male	0.89 (0.68‐1.18)	0.93 (0.71‐1.19)	1.23 (0.99‐1.54)	^g^1.39 (1.05-1.84)^**h**^	0.96 (0.75‐1.22)
Educational background (ref: bachelor’s degree and below)					
Master’s degree and above	0.90 (0.64‐1.27)	1.01 (0.75‐1.36)	0.94 (0.72‐1.22)	1.25 (0.89‐1.75)	0.90 (0.67‐1.20)
Hospital grade (ref: Level-II)					
Level-III	0.57 (0.39‐0.82)^i^	0.66 (0.48‐0.90)^i^	0.80 (0.61‐1.05)	0.56 (0.39‐0.81)^i^	0.86 (0.62‐1.20)
Professional title (ref: resident physician)					
Attending physician	1.06 (0.72‐1.54)	1.24 (0.88‐1.74)	1.18 (0.88‐1.60)	1.30 (0.87‐1.94)	0.85 (0.61‐1.19)
Chief physician	1.10 (0.63‐1.93)	0.93 (0.56‐1.52)	1.07 (0.69‐1.67)	0.89 (0.51‐1.58)	1.19 (0.73‐1.93)
Annual income level (ref: low)					
Middle	0.90 (0.65‐1.25)	0.99 (0.74‐1.32)	0.82 (0.63‐1.06)	0.72 (0.51‐1.01)	0.91 (0.69‐1.22)
High	0.92 (0.62‐1.36)	1.01 (0.72‐1.44)	0.78 (0.57‐1.07)	0.43 (0.29‐0.63)^i^	0.90 (0.64‐1.27)
Working hours (ref: ≤48 h/wk					
>48 h/wk	0.78 (0.58‐1.03)	0.74 (0.58-0.96)^h^	0.89 (0.71‐1.11)	0.60 (0.45‐0.80)^i^	1.04 (0.81‐1.34)
Night shifts (ref: ≤4 nights/time per month)					
>4 nights/time per month	0.86 (0.65‐1.15)	1.00 (0.78‐1.30)	1.02 (0.81‐1.28)	1.15 (0.86‐1.54)	0.86 (0.67‐1.11)
Health care working experience (ref: ≤10 years)					
>10 years	0.89 (0.57‐1.39)	1.07 (0.73‐1.59)	0.92 (0.65‐1.30)	1.15 (0.74‐1.79)	0.83 (0.57‐1.21)
Self-rated health status	1.09 (0.91‐1.29)	1.32 (1.12‐1.55)^i^	1.10 (0.95‐1.26)	1.23 (1.02-1.48)^h^	0.83 (0.70-0.97)^h^
Work Satisfaction Scale	1.04 (1.02‐1.06)^i^	1.14 (1.12‐1.16)^i^	1.10 (1.09‐1.12)^i^	1.16 (1.14‐1.18)^i^	0.91 (0.90‐0.93)^i^
Doctor-Patient Relationship Scale	1.08 (1.01‐1.16)^i^	0.86 (0.81‐0.92)^i^	0.94 (0.89‐0.99)^i^	0.77 (0.72‐0.82)^i^	1.25 (1.17‐1.33)^i^
Occupational Stress Scale	1.26 (1.22‐1.30)^i^	1.18 (1.15‐1.22)^i^	1.13 (1.11‐1.16)^i^	1.12 (1.08‐1.15)^i^	1.07 (1.04‐1.09)^i^

a Class 1: Reform-Adaptable group.

b Class 2: Negative group.

c Class 3: Neutral group.

d Class 4: Reform-Conservative group.

e Class 5: Positive group.

f OR: odds ratio.

g Bolded ORs indicate significance.

h *P*<.05.

i *P*<.01.

**Table 4.** Multinomial logistic regression results (Part B) examining the demographic and occupational predictors of membership in the 5 digital health technology adoption profiles among Chinese physicians (cross-sectional survey, 2023; N=4851).

Variable	Class 4^d^ vs Class 1^a^, ^f^OR (95% CI)	Class 4 vs Class 2^b^, OR (95% CI)	Class 4 vs Class 3^c^, OR (95% CI)	Class 3 vs Class 1, OR (95% CI)	Class 3 vs Class 2, OR (95% CI)
Age (years)	1.00 (0.97‐1.03)	0.99 (0.96‐1.01)	1.02 (0.99‐1.04)	0.99 (0.97‐1.01)	0.98 (0.96‐1.00)
Gender (ref: female)					
Male	0.64 (0.48‐0.85)^i^	0.67 (0.54‐0.84)^i^	0.89 (0.72‐1.10)	0.72 (0.58‐0.90)^i^	0.76 (0.64‐0.89)^i^
Educational background (ref: bachelor’s degree and below)					
Master’s degree and above	0.73 (0.52‐1.01)	0.80 (0.62‐1.05)	0.75 (0.59-0.96)^h^	0.97 (0.74‐1.25)	1.07 (0.89‐1.30)
Hospital grade (ref: Level-II)					
Level-III	1.01 (0.69‐1.48)	1.17 (0.86-1.59)	1.43 (1.08-1.89)^h^	0.71 (0.53-0.95)^h^	0.82 (0.66-1.01)
Professional title (ref: resident physician)					
Attending physician	0.81 (0.55‐1.21)	0.95 (0.68‐1.33)	0.91 (0.67‐1.23)	0.89 (0.67‐1.20)	1.05 (0.84‐1.31)
Chief physician	1.23 (0.70‐2.17)	1.03 (0.65‐1.63)	1.20 (0.79‐1.83)	1.02 (0.67‐1.59)	0.87 (0.63‐1.19)
Annual income level (ref: low)					
Middle	1.25 (0.90‐1.76)	1.38 (1.04-1.82)^h^	1.13 (0.87‐1.47)	1.10 (0.85‐1.43)	1.21 (1.00-1.46)^h^
High	2.15 (1.45‐3.18)^i^	2.38 (1.73‐3.26)^i^	1.83 (1.37‐2.45)^i^	1.17 (0.86‐1.59)	1.29 (1.03-1.62)^h^
Working hours (ref: ≤48 h/wk)					
>48 h/wk	1.30 (0.98-1.73)	1.25 (1.00-1.59)^h^	1.48 (1.19‐1.83)^i^	0.88 (0.70‐1.10)	0.84 (0.72-1.00)^h^
Night shifts (ref: ≤4 nights/time per month)					
>4 nights/time per month	0.75 (0.56‐1.01)	0.87 (0.69‐1.10)	0.88 (0.71‐1.10)	0.85 (0.68‐1.06)	0.99 (0.83‐1.17)
Health care working experience (ref: ≤10 years)					
>10 years	0.77 (0.50‐1.19)	0.93 (0.65‐1.33)	0.80 (0.58‐1.10)	0.97 (0.69‐1.36)	1.17 (0.91‐1.51)
Self-rated health status	0.89 (0.73‐1.07)	1.07 (0.92‐1.26)	0.89 (0.77‐1.03)	0.99 (0.86‐1.14)	1.20 (1.07‐1.34)^i^
Work Satisfaction Scale	0.89 (0.88‐0.91)^i^	0.98 (0.96‐0.99)^i^	0.95 (0.94‐0.96)^i^	0.94 (0.93‐0.95)^i^	1.03 (1.02‐1.04)^i^
Doctor-Patient Relationship Scale	1.40 (1.30‐1.51)^i^	1.12 (1.06‐1.19)^i^	1.23 (1.16‐1.29)^i^	1.14 (1.08‐1.21)^i^	0.92 (0.88‐0.96)^i^
Occupational Stress Scale	1.13 (1.09‐1.16)^i^	1.06 (1.03‐1.09)^i^	1.02 (1.01-1.04)^h^	1.11 (1.09‐1.14)^i^	1.04 (1.02‐1.06)^i^

a Class 1: Reform-Adaptable group.

b Class 2: Negative group.

c Class 3: Neutral group.

d Class 4: Reform-Conservative group.

e Class 5: Positive group.

f OR: odds ratio.

g Bolded ORs indicate significance.

h *P*<.05.

i *P*<.01.

Notably, several work-related patterns emerged from the analysis. Physicians from tertiary (Level-III) hospitals were significantly less likely to be in Class 5 than in Classes 1, 2, and 4 (OR 0.57, 95% CI 0.39‐0.82; OR 0.66, 95% CI 0.48‐0.90; and OR 0.56, 95% CI 0.29‐0.81, respectively; all *P*=.001), but more likely to be classified in Class 4 than in Class 3 (OR 1.43, 95% CI 1.08‐1.89; *P*=.008). Furthermore, higher income was strongly associated with membership in Class 4 compared with all other classes (vs Class 1: OR 2.15, 95% CI 1.45‐3.18; vs Class 2: OR 2.38, 95% CI 1.73‐3.26; vs Class 3: OR 1.83, 95% CI 1.37‐2.45; vs Class 5: OR 2.34, 95% CI 1.58‐3.48; all *P*=.001). Similarly, working more than 48 hours per week significantly increased the likelihood of belonging to Class 4 relative to Classes 2, 3, and 5 (OR 1.25, 95% CI 1.08‐1.89, *P*=.045; OR 1.48, 95% CI 1.19‐1.83, *P*=.001; OR 1.67, 95% CI 1.24‐2.22, *P*=.001, respectively). When compared with Class 2, members of Class 3 were more likely to have higher income levels (middle income: OR 1.21, 95% CI 1.00‐1.46, *P*=.047; high income: OR 1.29, 95% CI 1.03‐1.62, *P*=.03) yet less likely to work over 48 hours per week (OR 0.84, 95% CI 0.72‐1.00; *P*=.044).

Compared with Class 1, individuals with higher work satisfaction were more likely to belong to Class 5 (OR 1.04, 95% CI 1.02‐1.06), while those with lower work satisfaction showed greater probabilities of membership in Class 2 (OR 0.91, 95% CI 0.90‐0.93), Class 3 (OR 0.94, 95% CI 0.93‐0.95), and Class 4 (OR 0.89, 95% CI 0.88‐0.91). Higher occupational stress and more positive doctor-patient relationship perceptions were also significantly associated with membership in Classes 2, 3, 4, and 5 relative to Class 1 (all *P*=.001). When compared with Class 2, higher work satisfaction (OR 1.03, 95% CI 1.02‐1.04) and more negative doctor-patient relationship perceptions (OR 0.92, 95% CI 0.88‐0.96) predicted membership in Class 3, whereas lower work satisfaction (OR 0.98, 95% CI 0.96‐0.99) and more positive relationship perceptions (OR 1.12, 95% CI 1.06‐1.19) were associated with Class 4. Higher occupational stress elevated the probability of classification into both Class 3 (OR 1.04, 95% CI 1.03‐1.09) and Class 4 (OR 1.06, 95% CI 1.03‐1.09). Also, using Class 3 as the reference, higher work satisfaction reduced the likelihood of belonging to Class 4 (OR 0.95, 95% CI 0.94‐0.96), while more positive doctor-patient relationship perceptions increased it (OR 1.23, 95% CI 1.16‐1.29). All reported associations were statistically significant (*P*=.001).

Furthermore, compared with physicians in Classes 2, 3, and 4, those in Class 5 demonstrated distinct characteristics across 3 key domains. Specifically, Class 5 physicians showed significantly higher odds of severe occupational stress (OR range 1.12‐1.18; *P*=.001), reported greater work satisfaction (OR range 1.10‐1.16; *P*=.001), yet held less positive expectations regarding doctor-patient relationships (OR range 0.77‐0.94; *P*=.001; refer to Tables3  4 for details).

## Discussion

### Principal Findings

This study accomplished its 2 primary objectives by applying LPA to examine physicians’ adoption of DHTs. First, using a tripartite framework (Perceived Benefits, Adoption Barriers, and Behavioral Intention), the analysis identified 5 clinically meaningful profiles that moved beyond conventional classifications [32,33]: Reform-Adaptable (n=516, 10.64%), Negative (n=1003, 20.68%), Neutral (n=2276, 46.92%), Reform-Conservative (n=545, 11.23%), and Positive (n=511, 10.53%). Second, the analysis demonstrated that profile membership was systematically correlated with a range of key demographic and occupational factors, including gender, education, income, hospital tier, working hours, self-rated health, occupational stress, job satisfaction, and perceptions of doctor-patient relationships. This association confirms the substantial heterogeneity in DHT adoption among physicians. Given their pivotal role in implementing DHTs to enhance patient care [7], this divergence warrants attention and further investigation. By identifying the specific factors linked to each profile, our findings provide an empirical basis for developing tailored implementation strategies that account for these distinct physician subgroups.

In this study, we found that levels of occupational stress and work satisfaction differed significantly across the 5 latent profiles. Specifically, physicians reporting relatively high occupational stress alongside high work satisfaction were more likely to belong to Class 5 (Positive group), a profile characterized by greater perceived benefits and fewer adoption barriers regarding DHT implementation. To interpret this seemingly counterintuitive association, we used the Job Demands-Resources framework [34], which posits that high job demands can motivate the adoption of functional resources, including digital tools, to mitigate work pressure. Our findings support this mechanism: physicians in the Positive group indicated that DHTs contributed to improved work efficiency and better management of daily workloads, notably by facilitating remote consultations and streamlining follow-up processes. Rather than perceiving digital tools as additional burdens, these physicians used DHTs as strategic resources to maintain autonomy and reduce time-related pressures. This observation aligns with previous studies indicating that health care professionals under high workload demands often adopt efficiency-enhancing technologies, including automated electronic health records, to alleviate operational strain and prevent burnout [35].

Furthermore, we found that the combination of high stress and high job satisfaction likely reflects a subgroup of physicians who are highly engaged and adaptive. In our sample, those with greater work satisfaction (often stemming from institutional trust and personal adaptability) were generally more receptive to technological innovations promising improved efficiency, such as telemedicine systems [36]. Thus, our results suggest that, for certain physicians, occupational challenges may not inhibit but could even stimulate willingness to adopt practical digital solutions.

A notable divergence emerged between these findings and those of previous studies in the Western context [5,6], which identified physician age as a significant predictor of DHT adoption patterns. One plausible explanation may lie in the comprehensive integration of digital technologies within China’s health care system. The mandatory adoption of health codes during the COVID-19 pandemic and the widespread implementation of internet-based consultation systems may have reduced age-related digital disparities among physicians, diminishing the influence of online age as a distinguishing factor in DHT adoption. In addition, gender differences in DHT adoption patterns may reflect broader sociocultural dynamics within Chinese healthcare service systems. Female physicians—who comprised most of our sample—often bear disproportionate responsibilities for both clinical work and family care, which may limit their capacity to engage with new technologies that require additional training time. Previous studies suggest that women in healthcare settings, both in China and globally, tend to adopt a more cautious approach to technology adoption, prioritizing established practicality and reliability over novelty [5,37]. We also found that income level emerged as a significant predictor, likely reflecting structural aspects of China’s compensation system. Physicians in higher income brackets, often concentrated in specialized fields and tertiary hospitals, may perceive less economic incentive to adopt DHTs that could disrupt established workflows without immediate financial benefits. Conversely, physicians in lower-income segments might view DHTs as potential tools for improving efficiency and patient volume, thereby increasing earnings [6].

Furthermore, while no significant differences were observed across professional titles, physicians working in secondary hospitals demonstrated a more positive perception of DHTs, reporting higher perceived benefits and lower barriers to adoption compared with those in tertiary hospitals. This divergence may reflect systemic differences within China’s tiered health care system. Physicians in tertiary hospitals frequently face overwhelming clinical workloads and academic pressures, which may contribute to innovation fatigue despite their greater access to technological resources. In contrast, secondary hospital physicians may perceive DHTs as strategic tools for enhancing institutional competitiveness and addressing resource constraints through telemedicine collaborations with tertiary centers. These findings suggest that implementing targeted DHT strategies in secondary hospitals could be particularly effective for improving service quality and patient satisfaction. For example, the COVID-19 pandemic catalyzed the widespread deployment of teleconsultation platforms to ensure continuity of care [29,38]. Videoconferencing enables not only remote patient monitoring but also real-time supervision of clinical teams by specialists from tertiary hospitals [39]. Evidence shows that many DHTs provide affordable platforms for grassroots hospitals to collaborate with advanced medical centers. Through structured initiatives, including clinician exchanges, treatment protocol standardization, and technical assistance, DHTs have significantly improved the quality of care at primary health care institutions and are strongly aligned with China’s tiered health care policy objectives [5,40]. These technologies help bridge resource gaps and expand access to specialized care, particularly for patients in secondary hospitals. The distinct patterns identified in this study, such as the reduced role of physician age and heightened receptivity in secondary hospitals, are shaped by China’s specific health care policy landscape [37].

In fact, the national “Healthy China 2030” strategy explicitly prioritizes the integration of the internet, AI, and big data technologies throughout health care delivery [4]. This top-down mandate has catalyzed widespread institutional adoption of DHTs, creating an environment where exposure to digital tools is becoming universal. The rapid implementation of the health code system and telemedicine platforms during the COVID-19 pandemic, for instance, served as a form of nationwide digital training, which likely enhanced digital literacy among physicians of all demographic backgrounds and may have diminished conventional disparities associated with age [41]. Furthermore, as secondary hospitals are often direct targets of policy support and funding for digital capacity building, physicians in these settings report more positive perceptions of DHTs, viewing them as tools for professional advancement and better patient care. These findings may be generalizable to other health systems that use strong top-down digital integration policies and tiered care models, though local infrastructure and policy intensity would influence applicability.

Moreover, physicians with higher income levels, those working more than 48 hours per week, and those reporting more favorable doctor-patient relationships were more likely to belong to the Reform-Conservative group (Class 4), which perceived relatively low levels of both benefits and barriers associated with DHTs and maintained a conservative stance toward adoption. The association between more favorable doctor-patient relationships and membership in the Reform-Conservative group presents a theoretically intriguing paradox that merits elaboration. Rather than reducing DHT adoption, we believe this is because physicians with established positive patient relationships may perceive less need for DHTs that could potentially disrupt these carefully maintained interpersonal dynamics.

Within the Chinese health care context, where traditional relationship-centered models of care remain highly valued, physicians with strong patient relationships may view DHTs as potentially undermining the personal connection and trust they have cultivated. These physicians might perceive digital tools as introducing a layer of technological mediation into what they consider to be essentially human interactions, potentially diluting the emotional quality of care. Conversely, physicians experiencing challenges in patient communication might view DHTs as tools to enhance efficiency, standardize interactions, or overcome communication barriers, thus increasing their adoption motivation. This interpretation suggests that doctor-patient relationship quality operates not simply as a demographic variable but as a significant indicator of clinical satisfaction and practice style that consistently influences technology adoption decisions. Alternatively, this preference for traditional health care models may stem from the lack of observed improvements in service quality or efficiency post-DHT implementation in their settings, particularly among more clinically experienced physicians in demanding specialties such as neurosurgery, critical care, and emergency medicine. For these physicians, adapting complex workflows to incorporate DHTs may exacerbate feelings of burnout [17]. Similarly, in these demanding clinical environments, greater emphasis is placed on physicians’ technical competencies and their ability to deliver patient-centered health care services, which may consequently diminish their perceived need for DHTs [6].

In contrast, the Reform-Adaptable group demonstrates a risk-aware yet optimistic approach, recognizing significant benefits despite acknowledging implementation barriers, resulting in consistently high adoption intentions. This group exhibits greater flexibility, often engaging in selective adoption of technologies with clear clinical advantages and actively participating in pilot programs. Policy measures should accordingly diverge: for Reform-Conservative physicians, efforts must demonstrate fundamental value through evidence-based outcomes and success stories, whereas Reform-Adaptable physicians may benefit from targeted support, technical assistance, and roles as digital champions to address specific workflow integration concerns.

In addition, many health care systems have failed to fully operationalize the targeted intervention capabilities of AI and digital solutions [5]. Across numerous institutions, the fundamental requirements for successful DHT implementation remain challenging, as issues of service accessibility, standardized protocols, safety guarantees, and system reliability are still not adequately addressed [6]. As technological advancements progress and clinical feedback from various departments informs iterative improvements to DHT systems, emerging technological breakthroughs—alongside evolving patient attitudes toward digital health care—may gradually shift the perspectives of more conservative practitioners and facilitate wider DHT adoption [6].

Notably, approximately 31% of the physician cohort expressed significant concerns regarding DHT implementation barriers, particularly related to technological challenges, cybersecurity risks, increased workload, and potential negative impacts on patient experience. Consistent with previous comprehensive reviews [5,7,42,43], our study revealed that health care workers, regardless of the level of care or the specific technology involved, face recurring challenges related to infrastructure, technology, training, legal and ethical issues, time constraints, and workload increases. Furthermore, limitations on widespread DHT adoption are often rooted in health care workers’ anxiety about increased workload and disruptions to their established routines. This anxiety can contribute to professional burnout, which, in turn, threatens the long-term sustainability of these technologies [18,35]. These findings suggest that future development of DHTs should focus on thoughtfully integrating digital solutions with conventional clinical workflows to establish hybrid care delivery models that may help mitigate potential workload increases and burnout risks. To adequately address physicians’ concerns regarding DHT implementation, health care institutions should consider implementing tailored support systems. Specifically, customized training programs and continuing medical education initiatives designed to meet individual physicians’ competency needs and practice contexts could potentially reduce psychological barriers and facilitate more widespread, sustainable DHT adoption. Such personalized approaches may prove particularly valuable in addressing the varied adoption patterns identified in our study while maintaining clinical workflow integrity [5].

While this study focuses on Chinese physicians, our findings reveal both parallels and distinctions with international contexts. Consistent with European findings, skepticism regarding the clinical value and workflow impact of DHTs was prevalent [5,44]. However, unlike US research emphasizing financial incentives, DHT adoption in China was more influenced by institutional support [45,46]. Comparisons with other Asian settings showed similar hospital-level effects, though these were more pronounced in China’s policy-driven system. This suggests that while core adoption mechanisms may be universal, specific drivers remain culturally and systemically distinct [47].

### Implications for Policy and Practice

The heterogeneity observed in DHT adoption profiles highlights the limitations of relying solely on efficiency-driven models and underscores the necessity of multidimensional assessment frameworks to guide successful DHT implementation within health care systems. The key distinction between these profiles lies in their 3D evaluation: Perceived Benefits, Adoption Barriers, and Behavioral Intention. The Reform-Adaptable group, despite perceiving high barriers, maintains a high willingness due to strong benefit perception and requires barrier-specific support. In contrast, the Reform-Conservative group shows low willingness driven by limited perceived benefits, necessitating value demonstration interventions. This perceptual divergence calls for tailored implementation strategies rather than uniform policies. Profile-specific recommendations are provided in Section 3 of Multimedia Appendix 3.

Furthermore, this profiling framework enables the proactive management of systemic risks, such as workload intensification and burnout, particularly among overworked physicians (>48 hrs/wk) and conservative adopters. To ensure sustainable integration, especially in complex tertiary hospitals, health care systems must prioritize co-designed solutions that address critical implementation determinants such as interoperability, cybersecurity, and equitable workload redistribution. Consequently, policymakers can further support sustainable adoption by institutionalizing holistic adoption metrics that balance efficiency gains with medical workers’ well-being, ensuring that DHTs enhance rather than exacerbate pressures on the health care system. Consistent with the principles of the NASSS (Nonadoption, Abandonment, Scale-up, Spread, and Sustainability) framework principles, these strategies emphasize the need for context-adaptive implementation across technological, organizational, and professional dimensions, making them practical and scalable for long-term success [48].

### Strengths and Limitations

The current findings reveal heterogeneity among Chinese physicians, suggesting the potential value of tailored institutional measures and policies for DHT implementation. This study sought to introduce a person-centered analytical approach by using latent profile analysis, which moves beyond exclusive reliance on variable-centered methods to explore distinct typologies of physicians based on their multidimensional perceptions. This exploratory approach identified 5 potential subgroups, offering an alternative perspective for understanding adoption heterogeneity.

We developed and applied a preliminary 3D evaluation framework, encompassing perceived benefits, barriers, and overall willingness, to capture variations in adoption patterns. Furthermore, we examined how individual characteristics and occupational factors were associated with profile membership. The analyses indicated that the organizational context (eg, hospital tier) appeared to play a more prominent role than individual demographics in some profiles. These findings contribute to understanding physician acceptance within China’s policy environment and may offer a transferable methodological approach for examining technology adoption in other health care settings.

The typological framework itself represents a key innovation, offering a nuanced and actionable perspective for developing tailored interventions. For example, physicians in the Reform-Adaptable subgroup might benefit from barrier-reduction support, while those in the Reform-Conservative subgroup may require a clearer demonstration of technology value. The observed patterns around organizational determinants offer insights suggesting that national policy contexts might influence technology adoption pathways. By considering the characteristics of the different physician subgroups, health care administrators could explore ways to improve work environments, adjust workflows, and enhance DHT operational capabilities, potentially supporting physician engagement with DHT implementation.

Our study has several limitations that need to be acknowledged. First, the cross-sectional design of our study limits our ability to establish temporality and causality. While the selected evaluation indicators for DHT include both beneficial and adverse factors, future research must examine how health care professionals’ preferences evolve to support stronger causal inferences. Second, while this study benefits from a large sample size, its generalizability may be limited by the exclusive focus on physicians from Xi’an, China. Regions with different economic development levels, digital infrastructure, and policy implementation—both within China and globally—may demonstrate different adoption patterns. The digital health landscape varies significantly across health care systems in terms of funding, regulation, and technological readiness. However, the identified latent profiles and organizational influences reflect fundamental mechanisms that may transfer across similar contexts. Future research should validate these findings across diverse socioeconomic and cultural settings, particularly in rural areas and other countries with different health care models. Third, self-reported measures may involve social desirability bias, though anonymity was ensured. Future studies should include objective behavioral data.

### Future Research Directions

As noted in previous research, health care professionals’ work environments significantly influence their adoption of DHTs. Consequently, we propose the following specific research directions. First, qualitative approaches such as in-depth interviews and focus groups could elucidate the reasons for resistance, particularly among physician subgroups skeptical of or negative toward DHTs. Second, longitudinal and mixed methods studies are warranted to explore how workplace factors—including job stress and doctor-patient relationships—shape DHT preferences over time, and how such preferences may, in turn, shape perceptions of the work environment. Finally, future research should expand the evaluation of DHT adoption willingness by integrating motivational factors such as incentive structures, professional fulfillment, and opportunities for personal development. This would support the creation of more nuanced typologies of physician engagement and help identify context-dependent barriers and facilitators across varied clinical settings.

### Conclusion

This study used latent profile analysis to identify 5 distinct subgroups of Chinese physicians based on their perceptions of DHT adoption, providing a practical framework for designing precision interventions. While the profiles reveal considerable diversity in adoption attitudes, they also highlight unifying concerns about usability and professional autonomy that persist across all profiles. Our findings suggest divergent intervention pathways corresponding to these profiles. Reform-Adaptable physicians appear most likely to benefit from technical support and workflow integration, whereas Reform-Conservative physicians may respond better to compelling evidence of clinical value and peer success stories. These insights provide health care administrators and policymakers with empirically grounded guidance for developing tailored implementation strategies rather than relying on standardized approaches. Future research should validate the longitudinal stability of these profiles and assess tailored interventions through rigorous real-world trials. Ultimately, by embracing this nuanced understanding, health care systems can evolve from uniform implementation to precision enablement, thereby enhancing both the practical impact and responsible scalability of DHTs and addressing shared physician concerns.

## Supplementary material

10.2196/77840 Multimedia Appendix 1 Details of data resource and collection procedures.

10.2196/77840 Multimedia Appendix 2 The original questionnaire, including information on the scope of digital health technology (DHT) investigated in this study and the specific items within the DHT scale, as well as details of the Work Satisfaction Scale.

10.2196/77840 Multimedia Appendix 3 Additional analyses of digital health technology (DHT) adoption profiles, including correlation results, validation of the DHT scale, and targeted intervention recommendations.

10.2196/77840 Checklist 1 STROBE checklist for observational study.

## Abbreviations

aBIC: adjusted Bayesian information criterion
AI: artificial intelligence
AIC: Akaike information criterion
BIC: Bayesian information criterion
BLRT: bootstrap likelihood ratio
DHT: digital health technology
DPRQ-3: Doctor-Patient Relationship Questionnaire-3
LPA: latent profile analysis
NASSS: Nonadoption, Abandonment, Scale-up, Spread, and Sustainability
OR: odds ratio
STROBE: Strengthening the Reporting of Observational Studies in Epidemiology
UTAUT: Unified Theory of Acceptance and Use of Technology
