# Supplementary material for: Digital Health Technology Adoption Among Chinese Physicians: Latent Profile Analysis and Cross-Sectional Study
Source: J Med Internet Res. 2025 Nov 26;27:e77840. doi: 10.2196/77840 (PMC12661596; doi:10.2196/77840)
Supplement: Multimedia Appendix 1 [file jmir-v27-e77840-s001.docx]

**General Introduction**

**Participants and ethical declaration**

The Xi'an Medical Staff Survey (2023) employed a stratified PPS (probability proportional to size) cluster sampling design. Stratification included all licensed secondary/tertiary hospitals (n = 188) and primary healthcare facilities (THCs/CHCs; n = 237) in Xi'an. A 25% random selection by PPS was applied, with 47 hospitals and 59 primary healthcare facilities (PHFs) sampled, using total staff as the size measure. A census approach was used, inviting all current employees in the sampled facilities to participate. From the 42,904 healthcare workers who responded to the survey, 11,116 were physicians. Detailed sampling procedures are provided in Section 1. The study was approved by the Xi'an Jiaotong University Biomedical Ethics Committee (approval number XJTUAE2646), with electronic informed consent and institutional authorization obtained from all participants. This study was approved by the Biomedical Ethics Committee of Xi'an Jiaotong University (XJTUAE2646). Electronic informed consent was obtained from all participants, with institutional approval from the Xi'an Municipal Health Commission.

**Questionnaire design and topic setting**

Survey data were collected electronically using REDCap (version 15.2.1; Vanderbilt University), hosted by Xi'an Jiaotong University. The questionnaire was adapted from the physician module of China's Seventh National Health Services Survey, with the addition of a section on internet-based healthcare (Part C). The instrument consisted of seven sections: Part A, Basic Information (48 items); Part B, Health Status and Service Utilization (26 items); Part C, Internet-Based Healthcare (30 items); Part D, Work and Personal Characteristics (17 items); Part E, Workplace Attitudes (22 items); Part F, Practice Environment (9 items); and Part G, Perceived Changes (16 items). Validation rules in REDCap ensured data integrity. The survey questionnaire is available by contacting the corresponding author of this study. The survey instruments used to assess the principal outcome variables in this study are presented in Appendix A.

**Questionnaire quality control**

To ensure the integrity of the data, we implemented a comprehensive quality assurance framework throughout the survey process, including pre-survey, mid-survey, and post-survey stages. The online survey was administered via REDCap (version 15.2.1; Vanderbilt University). (1) Pre-survey measures included rigorous questionnaire design (adapted from the National Health Service Survey, with additional questions on internet-based healthcare), scientifically grounded sampling methods, a pilot study to refine the survey instrument, and standardized training for institutional liaisons. (2) During the survey, real-time data collection was closely monitored, and daily progress reports, along with feedback, were provided to participating facilities to encourage institutional liaisons to distribute the survey link to all eligible staff members, ensuring full institutional participation. (3) Post-survey, quality checks involved duplicate response detection (based on IP addresses and identical answers), time-to-complete thresholds, attention-check questions, and tests for logical consistency. After applying the quality control criteria (Section 2), 1,024 physician questionnaires were excluded, leaving 10,092 valid responses (90.8%, including 8,282 from hospitals and 1,810 from PHFs) (Table S1). Figure S1 provides a flowchart illustrating the number of participants screened, excluded, and ultimately included in the survey.

**Inclusion and exclusion criteria**

While the survey encompassed all healthcare workers, the current analysis focused exclusively on physicians meeting two criteria: (1) holding valid professional qualifications (licensed physician or assistant physician in Western/Traditional Chinese medicine), and (2) active engagement in clinical practice. Analyses included 10,092 physicians: 8,282 from hospitals and 1,810 from primary healthcare facilities (PHFs). The response and validity rates were 63.0% and 90.5% for hospitals, and 75.8% and 92.1% for PHFs, respectively. Physician-specific sampling weights (PSSW) were computed by combining institution-level weights (inverse of PPS sampling probabilities) and physician-to-staff ratios, with non-response adjustments stratified by gender, age, and professional title (Table S2). Final weights ensured representativeness of the target physician population (Section 3).

**Section 1: Sampling Strategy**

**1.1 Sampling of hospitals**

All secondary and tertiary hospitals in Xi’an as of the end of 2022 were included in the sampling frame. Data provided by the Xi’an Municipal Health Commission included each hospital’s name, total number of personnel, number of practicing (or assistant) physicians, and number of registered nurses. At the end of 2022, the hospital sampling frame comprised 188 institutions: 46 tertiary hospitals and 142 secondary hospitals. To ensure representativeness and feasibility, a sampling ratio of 25% was adopted, yielding a target sample of 47 hospitals. The sampling procedure followed standard PPS methodology, as outlined below:

(1) Sorting and Numbering: All 188 hospitals were ranked in descending order by total personnel and assigned serial numbers from 1 to 188. In cases of tied personnel counts across hospitals, a random order was applied among those with identical counts.

(2) Population Total (*N*): The total number of healthcare personnel across all 188 hospitals was calculated.

(3) Cumulative Size and Sampling Intervals: Each hospital’s cumulative personnel count range was computed. The sampling interval (*SI*) was then defined as *SI = N / 47*.

(4) Random Start and Selection: A random start value (*k*_1_) was generated using “*gen r = floor(runiform()*SI) + 1*” in StataMP 18. The 47 sample selections corresponded to:

$$Sample {point}_{i}=k_{1}+(i-1)\times SI, for i=1, 2, 3, \cdots, 47\cdot\cdot\cdot\cdot\cdot\cdot\cdot\cdot\cdot\cdot\cdot\cdot\cdot\cdot\cdot\cdot\cdot\cdot Equton (A1)$$

These values were mapped onto the cumulative size ranges to identify the selected hospitals.

**1.2 Sampling of primary healthcare facilities**

A separate sampling frame was constructed for primary healthcare institutions based on data from the Xi’an Municipal Health Commission. As of the end of 2022, there were 237 institutions in total–151 township health centers and 86 community health centers. A consistent sampling ratio of 25% was applied, yielding 59 selected institutions. The steps were analogous to those used for hospitals:

(1) Sorting and Numbering: All 237 primary healthcare institutions were sorted in descending order of personnel size and assigned serial numbers from 1 to 237. As above, random order was used to resolve ties.

(2) Population Total (*N′*): The total number of healthcare personnel across all primary healthcare institutions was computed.

(3) Cumulative Size and Sampling Intervals: Each institution’s cumulative personnel count range was calculated. The sampling interval was *SI′ = N / 59*.

(4) Random Start and Selection: A random seed (*k*_2_) was generated using the same procedure in Stata. The 59 sample points were determined by:

$$Sample {point}_{j}=k_{2}+(j-1)\times SI', for j=1, 2, 3, \cdots, 59\cdot\cdot\cdot\cdot\cdot\cdot\cdot\cdot\cdot\cdot\cdot\cdot\cdot\cdot\cdot\cdot\cdot\cdot Equton (A2)$$

Each point was matched to the institution whose cumulative size range it fell into.

**Section 2: Survey Quality Assurance Procedures**

**2.1 Pre-survey quality assurance**

(1) Questionnaire Development: The questionnaire was adapted from the “Seventh National Health Services Survey – Healthcare Worker Survey” with additional items on internet-based healthcare services. It included seven sections (Parts A–G) and a total of 168 items.

(2) Sampling Strategy: Described in detail in the “Sampling” section of the main text.

(3) Pilot Study: Conducted from Nov 1–3, 2023, involving 2 tertiary hospitals, 4 secondary hospitals, 10 township health centers (THCs), and 10 community health centers (CHCs), with 780 returned questionnaires (729 valid, validity rate: 93.5%). Based on findings, we refined question wording, layout, and survey procedures.

(4) Training of Survey Liaisons: Each sampled facility appointed a liaison, who received face-to-face training on survey objectives, procedures, REDCap system usage, and quality control. Communication was maintained via a dedicated WeChat group.

**2.2 Ongoing quality monitoring**

(1) Progress Monitoring: Real-time monitoring of questionnaire completion via REDCap allowed the research team to track institutional progress.

(2) Feedback Mechanism: Daily summaries (e.g., number of completed/valid questionnaires) were shared with institutional liaisons, who were encouraged to ensure full staff coverage. This dynamic feedback mechanism helped improve data completeness and timeliness.

**2.3 Post-survey data validation**

(1) Duplicate Response Detection: Responses from the same IP address with identical demographic information (Part A) were checked. If duplicates were found, only the latest response was retained.

(2) Response Duration Check: Based on the distribution of response times, questionnaires completed in under 5 minutes or over 120 minutes were flagged and excluded.

(3) Attention-Check Items: Two control items (Q11 and Q52) asked identical questions about institutional type but presented options in reversed order. Inconsistent answers led to exclusion.

(4) Logical Consistency: For example, the fixed income reported in Q18 had to be less than or equal to the total income in Q17. Responses violating such logic rules were excluded.

**2.4 Survey period and final sample**

The pilot study was conducted from Nov 1 to Nov 3, 2023. The formal survey took place from Nov 7 to Dec 8, 2023. A total of 42,904 responses were collected. After post-survey quality control, 39,944 valid questionnaires were retained (validity rate: 93.1%), including 10,092 physicians, 14,546 nurses, and 15,306 others (2,748 in public health, 4,652 in medical technology, 1,484 in pharmacy, and 6,422 in other roles).

**2.5 Data Quality Control and Missing Data Handling**

To ensure high data quality, we implemented a comprehensive quality control protocol. This included a pilot test with 814 health workers (achieving 93.5% compliance) and extensive training for liaison officers from 33 city-level hospitals and 9 county-level government departments on survey protocols and quality assurance. Our data validation process incorporated consistency checks (e.g., control questions), logic verification (e.g., years of service), outlier detection (e.g., age range), and completion time analysis.

From an initial pool of 8,617 responses, 3,766 records were excluded based on the following pre-defined criteria: 1) incomplete core demographic or DHT perception variables (n=283); 2) invalid or patterned responses (n=97); 3) excessively short completion times indicating non-serious engagement (n=46); and 4) employment at institutions where DHT had not been implemented or its status was unknown (n=3,431). These exclusions resulted in a final analytical sample of 4,851 complete cases.

To address the concern regarding missing data mechanism, we performed Little's Missing Completely at Random (MCAR) test. The results (χ² = 84.387, *df* = 81, *P*= 0.3765) supported the null hypothesis that the data were missing completely at random. Given the MCAR mechanism and the minimal proportion of incomplete cases (283 out of 8,617 initial responses), we determined that complete-case analysis would not introduce substantial bias while preserving the integrity of the latent profile analysis, which requires complete data for model estimation.

Little's MCAR test results

Number of observations sample = 4851

Number of variables = 13

Degrees of freedom = 81

chi2(12) = 84.387

Prob > chi2 = 0.3765

H0: Data are missing completely at random (MCAR)

**Section 3: Physician-Specific Sampling Weights**

Calculation Procedure: To ensure that the final analyses are representative of the physician population, we applied physician-specific sampling weights. These weights account for the complex, stratified probability-proportional-to-size (PPS) cluster sampling design and adjust for the inclusion of only the physician subset from each healthcare institution. The calculation was performed in the following steps:

**3.1 Hospital-level selection probability**

For each hospital *j* in the sampling frame (where *j* = 1, 2, …, *N* and *N* is the total number of hospitals), the selection probability *π_j_* was determined based on the total number of employed personnel *M_j_* (i.e., both clinical and non-clinical staff). Specifically, with a target of selecting *n* hospitals, the probability for hospital *j* is calculated as:

$$\pi_{j}=\frac{M_{j}}{\sum_{k=1}^{N} M_{K}}\times n\cdot\cdot\cdot\cdot\cdot\cdot\cdot\cdot\cdot\cdot\cdot\cdot\cdot\cdot\cdot\cdot\cdot\cdot\cdot\cdot\cdot\cdot\cdot\cdot\cdot\cdot\cdot\cdot\cdot\cdot\cdot\cdot\cdot\cdot\cdot\cdot\cdot\cdot\cdot\cdot\cdot\cdot\cdot\cdot\cdot\cdot\cdot\cdot\cdot\cdot\cdot\cdot\cdot\cdot\cdot\cdot\cdot\cdot\cdot\cdot\cdot\cdot\cdot\cdot\cdot\cdot\cdot\cdot\cdot\cdot\cdot\cdot\cdot\cdot\cdot\cdot\cdot\cdot\cdot\cdot\cdot\cdot\cdot\cdot\cdot\cdot\cdot\cdot\cdot Equton (A3)$$

The corresponding design weight for hospital *j* is then given by:

$$w_{j}^{hospital}=\frac{1}{\pi_{j}}\cdot\cdot\cdot\cdot\cdot\cdot\cdot\cdot\cdot\cdot\cdot\cdot\cdot\cdot\cdot\cdot\cdot\cdot\cdot\cdot\cdot\cdot\cdot\cdot\cdot\cdot\cdot\cdot\cdot\cdot\cdot\cdot\cdot\cdot\cdot\cdot\cdot\cdot\cdot\cdot\cdot\cdot\cdot\cdot\cdot\cdot\cdot\cdot\cdot\cdot\cdot\cdot\cdot\cdot\cdot\cdot\cdot\cdot\cdot\cdot\cdot\cdot\cdot\cdot\cdot\cdot\cdot\cdot\cdot\cdot\cdot\cdot\cdot\cdot\cdot\cdot\cdot\cdot\cdot\cdot\cdot\cdot\cdot\cdot\cdot\cdot\cdot\cdot\cdot\cdot\cdot\cdot\cdot\cdot\cdot Equton (A4)$$

**3.2 Determination of the Physician Proportion**

Within each hospital, the proportion of healthcare workers who are physicians was computed. Let *D_j_* represent the number of physicians in hospital *j*. The physician proportion is then:

$$p_{i}=\frac{D_{j}}{M_{j}}\cdot\cdot\cdot\cdot\cdot\cdot\cdot\cdot\cdot\cdot\cdot\cdot\cdot\cdot\cdot\cdot\cdot\cdot\cdot\cdot\cdot\cdot\cdot\cdot\cdot\cdot\cdot\cdot\cdot\cdot\cdot\cdot\cdot\cdot\cdot\cdot\cdot\cdot\cdot\cdot\cdot\cdot\cdot\cdot\cdot\cdot\cdot\cdot\cdot\cdot\cdot\cdot\cdot\cdot\cdot\cdot\cdot\cdot\cdot\cdot\cdot\cdot\cdot\cdot\cdot\cdot\cdot\cdot\cdot\cdot\cdot\cdot\cdot\cdot\cdot\cdot\cdot\cdot\cdot\cdot\cdot\cdot\cdot\cdot\cdot\cdot\cdot\cdot\cdot\cdot\cdot\cdot\cdot\cdot\cdot\cdot\cdot\cdot\cdot\cdot\cdot\cdot\cdot\cdot\cdot Equton (A5)$$

**3.3 Calculation of physician-specific weight**

The final physician-specific sampling weight for each respondent from hospital *j* combines the hospital-level design weight and the physician proportion:

$$w_{j}^{physician}=w_{j}^{hospital}\times p_{i}=\frac{1}{\pi_{j}}\times\frac{D_{j}}{M_{j}}\cdot\cdot\cdot\cdot\cdot\cdot\cdot\cdot\cdot\cdot\cdot\cdot\cdot\cdot\cdot\cdot\cdot\cdot\cdot\cdot\cdot\cdot\cdot\cdot\cdot\cdot\cdot\cdot\cdot\cdot\cdot\cdot\cdot\cdot\cdot\cdot\cdot\cdot\cdot\cdot\cdot\cdot\cdot\cdot\cdot\cdot\cdot\cdot\cdot\cdot\cdot\cdot\cdot\cdot Equton (A6)$$

This weight reflects both the probability of the institution's selection and the relative representation of physicians within that institution.

**3.4 Calculation of non-response adjustment weights**

To adjust for non-response bias, non-response adjustment weights were calculated by strata based on three key variables: gender, age group, and professional title. The stratified non-response adjustment weights account for differential response rates across these strata and ensure that the estimates reflect the characteristics of the overall physician population. The non-response adjustment weight for each stratum *s*, defined by gender, age group, and title, is calculated as:

$$w_{s}^{nonresponse}=\frac{N_{s}^{physician}}{R_{s}^{physician}}\cdot\cdot\cdot\cdot\cdot\cdot\cdot\cdot\cdot\cdot\cdot\cdot\cdot\cdot\cdot\cdot\cdot\cdot\cdot\cdot\cdot\cdot\cdot\cdot\cdot\cdot\cdot\cdot\cdot\cdot\cdot\cdot\cdot\cdot\cdot\cdot\cdot\cdot\cdot\cdot\cdot\cdot\cdot\cdot\cdot\cdot\cdot\cdot\cdot\cdot\cdot\cdot\cdot\cdot\cdot\cdot\cdot\cdot\cdot\cdot\cdot\cdot\cdot\cdot\cdot\cdot\cdot\cdot\cdot\cdot\cdot\cdot\cdot Equton (A7)$$

$N_{s}^{physician}$ is the total number of physicians in stratum *s* in the population (i.e., the total number of physicians in each gender, age group, and title combination); $R_{s}^{physician}$ is the number of responding physicians in stratum *s* (i.e., the number of physicians who completed the survey in each gender, age group, and title combination).

The final non-response adjusted weight for each physician in stratum sss is then combined with the physician-specific sampling weight as follows:

$$w_{j}^{adjusted}=w_{j}^{physician}\times w_{s}^{nonresponse}=w_{j}^{hospital}\times p_{i}\times w_{s}^{nonresponse}\cdot\cdot\cdot Equton (A8)$$

$w_{j}^{adjusted}$ is the final adjusted weight for physician jjj after considering both the sampling design and the non-response adjustment.

**3.5 Application in Analysis**

This final physician-specific weight ($w_{j}^{adjusted}$) is applied in all statistical models to ensure that the sample is representative of the entire physician population. It accounts for both the probability of selection and adjustments for non-response. Additionally, a 99th percentile trimming process was applied to handle extreme values, ensuring the weights are more robust. This final weight is then used in all subsequent statistical analyses, reflecting the true distribution of physicians across different strata.

**Table S1.** Survey participation metrics among physicians, nurses, and healthcare workers (HCWs).

|  | **Total**  **population**  **(N)** | **Eligible**  **sample** | **Responses** | **Response**  **rate (%)^b^** | **Weighted**  **response**  **rate (%)^c^** | **Valid**  **responses** | **Validity**  **rate (%)^d^** |
| --- | --- | --- | --- | --- | --- | --- | --- |
| **Hospitals** | | | | | | | |
| Physicians | 22,346 | 14,525 | 9,151 | 63.0 | 57.6 | 8,282 | 90.5 |
| Nurses | 36,906 | 21,861 | 14,519 | 66.4 | 61.6 | 13,546 | 93.3 |
| All HCWs^a^ | 88,264 | 51,759 | 34,811 | 67.3 | 62.4 | 32,548 | 93.6 |
| **Primary healthcare facilities** | | | | | | | |
| Physicians | 3,779 | 2,594 | 1,965 | 75.8 | 73.6 | 1,810 | 92.1 |
| Nurses | 3,733 | 1,453 | 1,047 | 72.1 | 69.3 | 982 | 93.8 |
| All HCWs^a^ | 16,090 | 9,988 | 7,811 | 78.2 | 75.7 | 7,366 | 94.3 |
| **All healthcare facilities** | | | | | | | |
| Physicians | 26,125 | 17,119 | 11,116 | 64.9 | 62.1 | 10,092 | 90.8 |
| Nurses | 40,639 | 23,314 | 15,566 | 66.8 | 64.2 | 14,528 | 93.3 |
| All HCWs^a^ | 104,354 | 61,747 | 42,622 | 69.0 | 66.9 | 39,944 | 93.7 |

**Note:** ^a^ HCWs indicates healthcare workers. ^b^ Response rate = Responses / Eligible sample. ^C^ Weighted response rate = Σ(sampling weight × responses) / Σ(sampling weight × eligible sample). ^d^ Validity rate = Valid responses / Responses.

**Table S2.** Characteristics of physicians in valid survey samples vs. target physician population.

|  | **Hospitals** | | | |  | **Primary healthcare facilities** | | | |
| --- | --- | --- | --- | --- | --- | --- | --- | --- | --- |
|  | **Valid**  **response (%)** | **Target**  **(%)** | **Pearson χ²** | ***P*-**  **valve** |  | **Valid**  **response (%)** | **Target**  **(%)** | **Pearson χ²** | ***P*-**  **valve** |
| **Sex** |  |  | 92.45 | <.001 |  |  |  | 5.33 | .021 |
| Female | 60.6 | 55.9 |  |  |  | 52.0 | 54.9 |  |  |
| Male | 39.4 | 44.1 |  |  |  | 48.0 | 45.1 |  |  |
| **Age group** |  |  | 1,020.64 | <.001 |  |  |  | 72.14 | <.001 |
| ≤34 years | 41.6 | 31.0 |  |  |  | 23.2 | 26.0 |  |  |
| 35–44 years | 35.2 | 35.0 |  |  |  | 24.4 | 29.3 |  |  |
| ≥45years | 23.3 | 34.0 |  |  |  | 52.5 | 44.7 |  |  |
| **Title** |  |  | 1,292.50 | <.001 |  |  |  | 102.47 | <.001 |
| Primary | 29.8 | 35.0 |  |  |  | 64.7 | 59.8 |  |  |
| Intermediate | 41.4 | 34.8 |  |  |  | 29.2 | 27.9 |  |  |
| Associate senior | 22.1 | 20.0 |  |  |  | 6.0 | 10.2 |  |  |
| Senior | 6.7 | 10.2 |  |  |  | 0.2 | 2.1 |  |  |

**Note:** This table compares the characteristics of 10,092 physicians with valid responses to the target physician population of 26,125 individuals to assess sample representativeness.


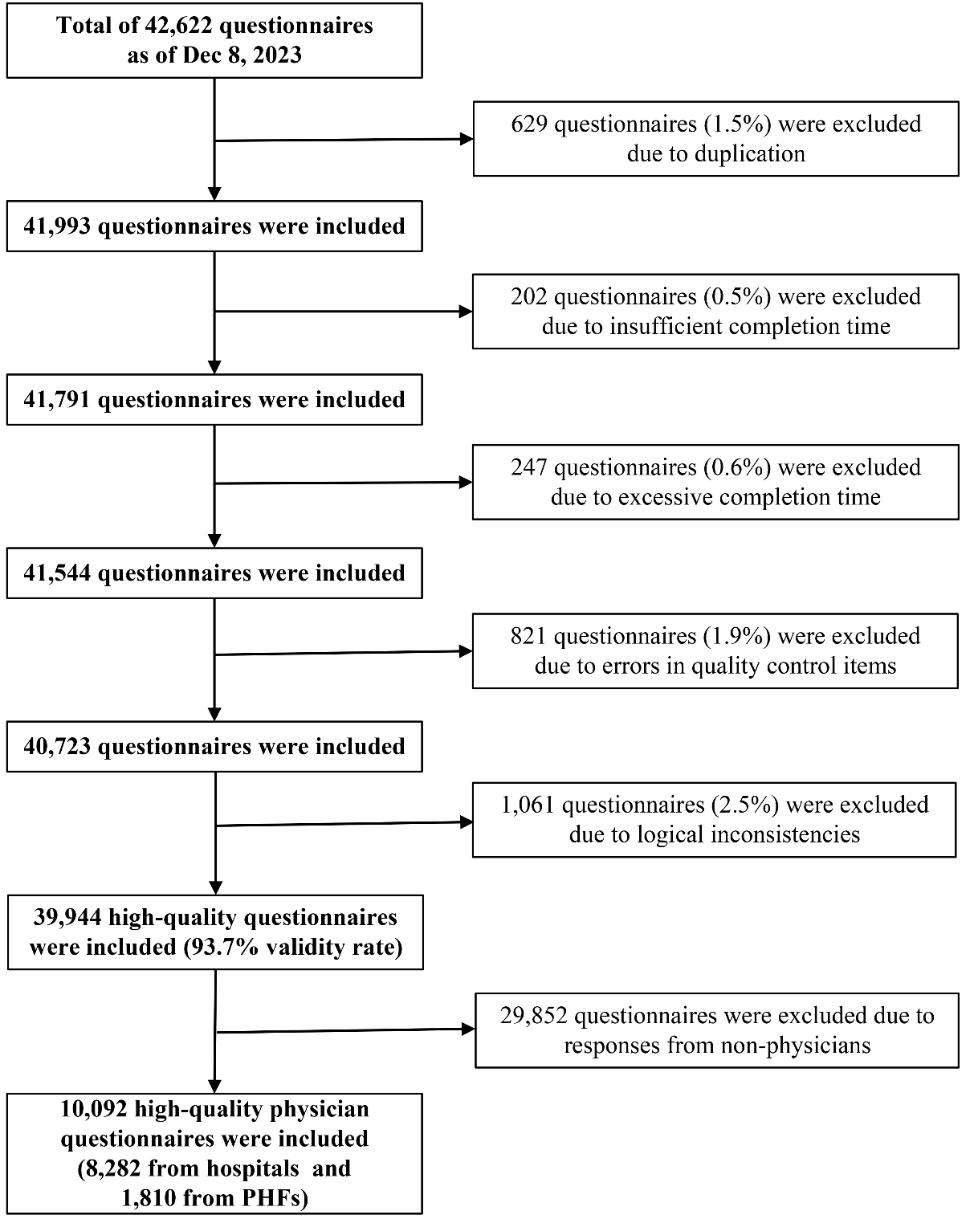


**Figure S1.** Flowchart of study participants.

Note: PHFs indicate primary healthcare facilities.
