# Supplementary material for: Digital Health Technology Adoption Among Chinese Physicians: Latent Profile Analysis and Cross-Sectional Study
Source: J Med Internet Res. 2025 Nov 26;27:e77840. doi: 10.2196/77840 (PMC12661596; doi:10.2196/77840)
Supplement: Multimedia Appendix 2 [file jmir-v27-e77840-s002.docx]

Study Questionnaire (Main Outcome Variables)

# Part A: Digital health technology adaptation

**1. “Which of the following healthcare services are supported by digital health technologies within your organization? (Select all that apply)”**

(1) Health monitoring

(2) Online consultations

(3) Post-treatment follow-ups

(4) Rehabilitation guidance

(5) Online-to-offline referrals

(6) Telemedicine services

(7) Medical insurance settlement

(8) Test report inquiries

(9) Home nursing care

(10) Health checkup and appointment booking

(11) Health education

(12) Electronic health records

(13) Data sharing

(14) Emergency medical services

(15) Medication consultation

(16) Drug delivery services

(17) Traditional Chinese medicine services

(18) Vaccination services

(19) Public health emergency alerts

(20) Others

(21) Not Applicable

**2. Which of the following digital health technology-based healthcare programs has your organization implemented? (Select all that apply).**

(1) Provided training on internet-based healthcare (theory, technology, online

services, and doctor-patient communication)

(2) Provided technical support for internet-based healthcare

(3) Provided hardware equipment for internet-based healthcare (e.g., computers)

(4) Incorporated internet-based healthcare into performance evaluations

(5) None of the above

(6) Not aware

**3. Perceptions of digital healthcare technology adoption.**

Regardless of your prior experience with internet-based healthcare, please rate your level of agreement with the following statements on a scale of 1 to 5 (1=strongly agree, 2=somewhat agree, 3=neutral, 4=somewhat disagree, and 5=strongly disagree).

| **Domains** | **Indicator** | **Item** | **Questionnaire** |
| --- | --- | --- | --- |
| Perceived Benefits | Diagnosis/  Treatment Quality | **Q1** | I believe internet-based healthcare helps patients express their needs more freely and allows me to better understand their conditions and required services in advance. |
|  |  | **Q2** | I believe internet-based healthcare enhances the quality of multidisciplinary consultations. |
|  | Patient Trust-Satisfaction | **Q3** | I find it difficult to establish trust with patients through internet-based healthcare. |
|  |  | **Q4** | I believe using internet-based healthcare increases patients’ recognition and satisfaction with my services. |
|  | Error Rate Reduction | **Q5** | I believe business information systems in internet-based healthcare can effectively minimize medical errors. |
|  |  | **Q6** | I believe internet-based healthcare improves referral efficiency and prevents referral disconnection. |
|  |  | **Q7** | I believe internet-based healthcare fosters collaboration among healthcare professionals. |
|  | Income Increase | **Q8** | I believe internet-based healthcare can significantly increase my income. |
| Adoption  Barriers | Technical Barriers | **Q9** | Compared to in-person services, I find providing internet-based healthcare services requires more complex procedures. |
|  |  | **Q10** | I am confident in handling common operational issues when using internet-based healthcare. |
|  | Cybersecurity Risks | **Q11** | I am concerned about security risks such as personal information, account passwords, and medical records being stolen when using internet-based healthcare. |
|  | Workload Increase | **Q12** | I worry that internet-based healthcare will take up too much of my personal and work time, increasing my workload. |
|  | Patient Experience Reduction | **Q13** | I worry that not being able to see patients in person when using internet-based healthcare increases medical risks and doctor-patient disputes. |
| Behavioral Intention | Overall willingness | **Q14** | If possible, I am willing to try or continue using internet-based healthcare in my work. |

# Part B: Work Satisfaction

This part explores healthcare professionals' perceptions of their work. It includes 10items, each rated on a 6-point Likert scale (1 = Strongly Agree, 2 = Somewhat Agree, 3 = Slightly Agree, 4 = Slightly Disagree, 5 = Somewhat Disagree, 6 = Strongly Disagree) (see Appendix Table B3).

**Work Satisfaction**

| **Dimension** | **Item** | **Questionnaire** |
| --- | --- | --- |
| **Overall job satisfaction** | **Q1** | Overall, I am very satisfied with my current job. |
| **Satisfaction with colleagues** | **Q2** | I am satisfied with my colleagues. |
| **Expected income** | **Q3** | Considering my skills and efforts, I am satisfied with my income. |
| **Leadership** | **Q4** | I am satisfied with my supervisor. |
| **Working facilities** | **Q5** | I am satisfied with my workplace conditions and equipment. |
| **Promotion prospects** | **Q6** | I am satisfied with my career advancement and promotion opportunities. |
| **Internal management** | **Q7** | I am satisfied with my institution’s management. |
| **Welfare benefits** | **Q8** | I am satisfied with the benefits and welfare provided by my job. |
| **Training opportunities** | **Q9** | I am satisfied with the training opportunities offered at work. |
| **Opportunity for skill use** | **Q10** | I am satisfied with the opportunities to utilize my skills at work. |
