# Supplementary material for: Digital Health Technology Adoption Among Chinese Physicians: Latent Profile Analysis and Cross-Sectional Study
Source: J Med Internet Res. 2025 Nov 26;27:e77840. doi: 10.2196/77840 (PMC12661596; doi:10.2196/77840)
Supplement: Multimedia Appendix 3 [file jmir-v27-e77840-s003.doc]

**Section 1**

**Table S1.** Pearson correlation coefficients assessing relationships between key study variables: digital health technology (DHT) adoption indicators, work satisfaction, doctor-patient relationship, and occupational stress in a multi-center sample of Chinese physicians (n=4,851).

| Variables | 1 | 2 | 3 | 4 | 5 | 6 | 7 | 8 | 9 | 10 | 11 | 12 |
| --- | --- | --- | --- | --- | --- | --- | --- | --- | --- | --- | --- | --- |
| 1. Diagnosis Treatment Quality | 1 |  |  |  |  |  |  |  |  |  |  |  |
| 1. Patient Trust-Satisfaction | 0.373^c^ | 1 |  |  |  |  |  |  |  |  |  |  |
| 1. Error Rate Reduction | 0.773^c^ | 0.351^c^ | 1 |  |  |  |  |  |  |  |  |  |
| 1. Income Increase | 0.347^c^ | 0.076^c^ | 0.444^c^ | 1 |  |  |  |  |  |  |  |  |
| 1. Technical Barriers | 0.302^c^ | -0.110^c^ | 0.348^c^ | 0.285^c^ | 1 |  |  |  |  |  |  |  |
| 1. Cybersecurity Risks | 0.195^c^ | -0.189^c^ | 0.198^c^ | 0.221^c^ | 0.380^c^ | 1 |  |  |  |  |  |  |
| 1. Workload Increase | 0.091^c^ | -0.275^c^ | 0.096^c^ | 0.177^c^ | 0.393^c^ | 0.633^c^ | 1 |  |  |  |  |  |
| 1. Patient Experience Reduction | 0.150^c^ | -0.262^c^ | 0.139^c^ | 0.129^c^ | 0.405^c^ | 0.668^c^ | 0.628^c^ | 1 |  |  |  |  |
| 1. Overall Willingness | 0.617^c^ | 0.336^c^ | 0.628^c^ | 0.494^c^ | 0.257^c^ | 0.136^c^ | 0.031^b^ | 0.086^c^ | 1 |  |  |  |
| 1. Doctor-patient Relationship | -0.251^c^ | -0.181^c^ | -0.262^c^ | -0.180^c^ | -0.063^c^ | 0.067^c^ | 0.111^c^ | 0.070^c^ | -0.251^c^ | 1 |  |  |
| 1. Work Satisfaction | 0.393^c^ | 0.200^c^ | 0.408^c^ | 0.299^c^ | 0.149^c^ | 0.018 | -0.064^c^ | -0.001 | 0.350^c^ | -0.393^c^ | 1 |  |
| 12. Occupational Stress | -0.046^c^ | -0.159^c^ | -0.011 | 0.108^c^ | 0.180^c^ | 0.243^c^ | 0.326^c^ | 0.217^c^ | -0.032^b^ | 0.238^c^ | -0.128^c^ | 1 |

Note: ^a^ *P < .05;* ^b^ *P < .01.* ^c^ *P < .001.*

**Table S2.** Profile-specific means and standard deviations for the three core DHT evaluation dimensions (perceived benefits, adoption barriers, behavioral intention) used in the latent profile analysis to identify five distinct subgroups of Chinese physicians (n=4,851).

| Indicator Variables | Class1 | Class2 | Class3 | Class4 | Class5 | *P* | η2 | Bonferroni post-hoc test |
| --- | --- | --- | --- | --- | --- | --- | --- | --- |
|  | N=516 | N=1003 | N=2276 | N=545 | N=511 |  |  |  |
|  | Mean (SD) | Mean (SD) | Mean (SD) | Mean (SD) | Mean (SD) |  |  |  |
| Diagnosis Treatment Quality | 4.69 (0.45) | 3.16 (0.62) | 4.13 (0.47) | 3.25 (0.65) | 4.94 (0.21) | <.001 | 0.584 | a>b,c,d; a<e; b<c ,d,e;  c>d; c<e; d<e |
| Patient Trust Satisfaction | 3.99 (0.78) | 3.02 (0.41) | 3.26 (0.55) | 2.50 (0.55) | 3.23 (0.62) | <.001 | 0.286 | a>b,c,d,e; b<c,e; b>d;  c>d; d<e |
| Error Rate Reduction | 4.48 (0.55) | 2.96 (0.51) | 3.88 (0.47) | 2.92 (0.64) | 4.90 (0.28) | <.001 | 0.627 | 1. b,c,d; a<e; b<c,e; c>d; 2. c<e; d<e |
| Increased Income | 3.07 (1.16) | 2.74 (0.62) | 3.15 (0.81) | 2.18 (0.85) | 4.47 (0.93) | <.001 | 0.314 | a>b,d; a<e; b>d; b<c,e;  c>d; c<e; d<e |
| Technical Barriers | 3.25 (1.36) | 2.99 (0.59) | 3.68 (0.75) | 3.85 (0.87) | 4.82 (0.52) | <.001 | 0.286 | a>b ; a<c,d, e; b<c,d,e;  c<d,e; d<e |
| Cybersecurity Risks | 2.29 (0.93) | 2.88 (0.56) | 3.90 (0.62) | 4.24 (0.78) | 4.90 (0.32) | <.001 | 0.570 | a<b,c,d,e; b<c.d.e;  c<d,e; d<e |
| Workload Increase | 2.19 (0.87) | 2.97 (0.57) | 3.75 (0.69) | 4.40 (0.67) | 4.70 (0.70) | <.001 | 0.515 | a<b,c,d,e; b<c.d.e;  c<d,e; d<e |
| Patient Experience Reduction | 2.47 (0.99) | 3.06 (0.66) | 3.98 (0.59) | 4.62 (0.52) | 4.88 (0.42) | <.001 | 0.559 | a<b,c,d,e; b<c,d,e;  c<d,e; d<e |
| Overall Willingness | 4.46 (0.61) | 2.99 (0.57) | 3.80 (0.58) | 2.72 (0.84) | 4.88 (0.38) | <.001 | 0.528 | a>b,c,d; a<e; b<c,e; b>d;  c>d; c, d<e |

Note: Class 1: Reform-Adaptable Group; Class 2: Negative Group; Class 3: Neutral Subgroup; Class 4: Reform-Conservative Group; Class 5: Positive Group.

a, b, c, d, e showed the results of the Bonferroni post-hoc tests, and there is a statistically significant difference between two. e.g., for Diagnosis Treatment Quality a>b,c, d; a<e;’ means that Class 1 has a mean score that is higher than Class 2,3 and 5 but lower than Class 5.

**Table S3.** Average Latent Class Probabilities for Most Likely Latent Class Membership (Row) by Latent Class (Column) among Physicians in the Digital Health Technology Adoption Analysis (n=4,851)

| *Latent Class* | *Class1* | *Class2* | *Class3* | *Class4* | *Class5* |
| --- | --- | --- | --- | --- | --- |
|  | *N=516* | *N=1003* | *N=2276* | *N=545* | *N=511* |
| Reform-Adaptable Group | 0.935 | 0.017 | 0.047 | 0 | 0.001 |
| Negative Group | 0.006 | 0.928 | 0.048 | 0.018 | 0 |
| Neutral Subgroup | 0.012 | 0.030 | 0.922 | 0.023 | 0.013 |
| Reform-Conservative Group | 0 | 0.041 | 0.078 | 0.881 | 0 |
| Positive Group | 0.001 | 0 | 0.040 | 0 | 0.959 |

Note: his table displays the average latent class probabilities for the most likely latent class membership (row) by latent class (column). Values on the diagonal (in bold) represent the average probability that an individual classified into a given latent class truly belongs to that class, which indicates classification accuracy. Off-diagonal values represent the probabilities of misclassification.

**Table S4.** Variance Inflation Factor values for predictors of physician digital health technology adoption profiles in China (n=4851).

| Variables | VIF | 1/ VIF | Multicollinearity  Concern |
| --- | --- | --- | --- |
| Age | 3.37 | 0.39 | No Concern |
| Gender (Ref=Female) |  | | |
| Male | 1.08 | 0.93 | No Concern |
| Hospital grade (Ref Level-II ) |  |  |  |
| Level-III | 1.42 | 0.70 | No Concern |
| Educational Background (Ref=Bachelor's degree and below) |  |  |  |
| Master's degree and above | 1.60 | 0.65 | No Concern |
| Professional title (Ref=Resident physician) |  | |  |
| Attending physician | 2.02 | 0.49 | No Concern |
| Chief physician | 4.15 | 0.26 | No Concern |
| Annual income level (Ref=Low) |  |  |  |
| Middle | 1.47 | 0.68 | No Concern |
| High | 1.86 | 0.54 | No Concern |
| Weekly work hours (Ref= ≤48h per week) |  | |  |
| >48 per week | 1.18 | 0.91 | No Concern |
| Monthly night shifts (Ref= ≤ 4 night/time) |  | |  |
| > 4 night/time | 1.28 | 0.82 | No Concern |
| Healthcare working experience (Ref= ≤ 10 years) |  | |  |
| >10 years | 2.77 | 0.36 | No Concern |
| Self-rated health status | 1.30 | 0.77 | No Concern |
| Work Satisfaction Scale | 1.25 | 0.80 | No Concern |
| Doctor-patient relationship Scale | 1.28 | 0.78 | No Concern |
| Occupational Stress Scale | 1.24 | 0.81 | No Concern |
| Mean VIF | 1.82 |  | No Concern |

**Section 2**

The Digital Health Technology Adoption Scale was developed through a multi-stage process to ensure its validity and reliability. First, a comprehensive literature review was conducted to identify potential items and establish a theoretical foundation based on established technology acceptance models and prior research on DHT adoption [[[1]](#footnote-2),[[2]](#footnote-3)]. This initial pool of items was then evaluated by a panel of five experts, including two health informatics specialists, two senior clinicians with experience in DHT implementation, and one methodology expert. The panel assessed each item for content validity, relevance, clarity, and comprehensiveness using a structured evaluation form. Items with low relevance scores (<0.78 on the content validity index) or ambiguous wording were revised or eliminated.

Following expert validation, the revised scale underwent pilot testing with a sample of 729 medical staff members (780 returned questionnaires, with 729 valid responses, yielding a validity rate of 93.5%) from various specialties and hospital levels. Participants completed the scale and provided feedback on item clarity, comprehension, and overall appropriateness through structured interviews. The pilot testing confirmed that the items were generally understood as intended and could be completed within an appropriate time frame (approximately 8-10 minutes). Based on pilot feedback, minor wording adjustments were made to enhance clarity and reduce ambiguity.

The final scale demonstrated excellent internal consistency in the main study sample, with a Cronbach’s alpha of 0.88 for the overall scale. Subscale reliability was also strong: perceived benefits (α=0.866) and adoption barriers (α=0.793). The behavioral intention item demonstrated appropriate discriminatory power in subsequent analyses. Detailed information regarding the validity of the scale is provided in Table S4.

**Table S5**. Assessment of the validity and reliability of the Digital Health Technology Adoption Scale in a national sample of Chinese physicians (Cross-sectional survey, 2023; N=4851), including factor loadings, composite reliability, and average variance extracted.

| Domain | Item | Average variance extracted | Composite reliability | Cronbach’s α coefficient |
| --- | --- | --- | --- | --- |
| Perceived Benefits | improved diagnostic and treatment quality | 0.642 | 0.821 | 0.866 |
|  | enhanced patient trust and satisfaction |  |  |  |
|  | error rate reduction |  |  |  |
|  | increased income |  |  |  |
| Adoption Barriers | technical barriers | 0.532 | 0.803 | 0.793 |
|  | cybersecurity risks |  |  |  |
|  | workload increase |  |  |  |
|  | patient experience reduction |  |  |  |
| Behavioral Intention | overall willingness | - | - | - |
| Overall Scale | | 0.620 | 0.856 | 0.881 |

**Section 3**

Our findings demonstrate the necessity of moving beyond one-size-fits-all DHT implementation strategies. Based on the distinct characteristics of each DHT adoption profile and related literature, we propose the following targeted interventions:

For the Reform-Adaptable group (Class 1), who recognize both benefits and barriers and show measured openness, strategies should engage them as co-designers and implementation partners. Effective approaches include: (1) integrating them into pre-implementation pilot-testing phases and structured feedback cycles to refine DHT usability; (2) appointing them as facilitators or group leaders in peer-training initiatives to leverage their balanced perspective; and (3) adjusting clinical schedules or reducing administrative burdens to compensate for the extra time required for DHT integration and problem-solving.

For the Negative group (Class 2), who exhibit pervasive skepticism toward DHTs, interventions must address both competence and confidence barriers through structured support programs. These should include: (1) mandatory, hands-on technical training sessions focused on overcoming specific digital literacy gaps and minimizing operational friction; (2) establishing formal mentorship programs that pair them with Positive group (Class 5) for sustained peer support and encouragement; and (3) implementing institutional mechanisms such as protected learning time, certifications, or monetary incentives to acknowledge and reward engagement with DHT training.

For the Neutral group (Class 3), whose adoption decisions are heavily influenced by contextual and social factors, strategies should foster a supportive environment that reduces uncertainty. Recommendations include: (1) securing visible endorsements from respected clinical leaders within their department who can model DHT engagement; (2) developing clear, step-by-step institutional protocols that embed DHT use into existing clinical workflows with minimal disruption; and (3) systematically collecting and sharing testimonials and usage data from colleagues who have successfully incorporated DHTs into practice.

For the Reform-Conservative group (Class 4), which remains skeptical of the fundamental value proposition of DHTs, policy efforts should prioritize evidence-based value demonstration. Concrete interventions include: (1) organizing specialty-specific case presentations by early adopters with comparable clinical backgrounds to illustrate relevant use cases; (2) disseminating rigorously collected data on DHTs’ impacts on clinically meaningful outcomes, such as diagnostic accuracy, treatment efficacy, or patient follow-up adherence; and (3) providing transparent, specialty-specific cost-benefit analyses that outline time investments, workflow adjustments, and potential returns in terms of efficiency or revenue.

For the Positive group (Class 5), who are enthusiastic early adopters, policies should formally recognize and harness their advocacy. This can be achieved by: (1) officially designating them as departmental digital health champions with defined roles and responsibilities; (2) providing dedicated resources (e.g., release time, small budgets) to support their peer-mentoring activities; and (3) including them in hospital-level technology procurement and implementation strategy committees to ensure frontline provider perspectives inform decision-making.

These tailored approaches recognize that effective DHT implementation requires addressing the specific concerns, motivations, and capabilities of each physician subgroup.

1. [?] Kane H, Gourret Baumgart J, El-Hage W, Deloyer J, Maes C, et al. Opportunities and Challenges for Professionals in Psychiatry and Mental Health Care Using Digital Technologies During the COVID-19 Pandemic: Systematic Review. JMIR Hum Factors. 2022;9(1):e30359. doi: 10.2196/30359. [↑](#footnote-ref-2)
2. [?] Schreiweis B, Pobiruchin M, Strotbaum V, Suleder J, Wiesner M, Bergh B. Barriers and Facilitators to the Implementation of eHealth Services: Systematic Literature Analysis. J Med Internet Res. 2019;21(11):e14197. doi: 10.2196/14197. [↑](#footnote-ref-3)
